# BH3-mimetics: recent developments in cancer therapy

**DOI:** 10.1186/s13046-021-02157-5

**Published:** 2021-11-09

**Authors:** Paul A. Townsend, Maria V. Kozhevnikova, Olivier N. F. Cexus, Andrey A. Zamyatnin, Surinder M. Soond

**Affiliations:** 1 University of Surrey, Guildford, UK; 2 Sechenov First Moscow State Medical University, Moscow, Russian Federation; 3 University of Manchester, Manchester, UK; 4 Lomonosov Moscow State University, Moscow, Russian Federation; 5 Sirius University of Science and Technology, Sochi, Russian Federation

**Keywords:** Apoptosis, BH-3 mimetics, PUMA-mimetics, Smac-mimetics, Bcl-xL-mimetics, Noxa-mimetics, Mcl1-mimetics, Nanoparticles

## Abstract

The hopeful outcomes from 30 years of research in BH3-mimetics have indeed served a number of solid paradigms for targeting intermediates from the apoptosis pathway in a variety of diseased states. Not only have such rational approaches in drug design yielded several key therapeutics, such outputs have also offered insights into the integrated mechanistic aspects of basic and clinical research at the genetics level for the future. In no other area of medical research have the effects of such work been felt, than in cancer research, through targeting the BAX-Bcl-2 protein-protein interactions. With these promising outputs in mind, several mimetics, and their potential therapeutic applications, have also been developed for several other pathological conditions, such as cardiovascular disease and tissue fibrosis, thus highlighting the universal importance of the intrinsic arm of the apoptosis pathway and its input to general tissue homeostasis. Considering such recent developments, and in a field that has generated so much scientific interest, we take stock of how the broadening area of BH3-mimetics has developed and diversified, with a focus on their uses in single and combined cancer treatment regimens and recently explored therapeutic delivery methods that may aid the development of future therapeutics of this nature.

## Background

Harnessing the potential of apoptosis, as a strategic approach for the eradication of cancer cells, has been an area of intense activity over the last 30 years, ranging from the implementation of death inducing ligands and therapeutics, to engineering synergistic chemical antagonists [1, 2]. What originally started as research aimed at unveiling the mechanistic input of extrinsic and intrinsic signaling pathways in cell demise, has indeed developed towards how such pathways can be therapeutically exploited as the findings have transitioned from a basic- to applied- research setting. Underpinning such developments have unambiguously relied on defining an ever-growing number of molecular signaling networks and their detailed regulatory crosstalk in defining potential axes of regulation that may be amenable for therapeutic intervention [3].

Herein, one critical regulatory event central to triggering apoptosis is the activation of Mitochondrial Outer Membrane Polarization (MOMP) and is the cornerstone of intrinsic pathway activation. How this is achieved at the molecular level, and what factors regulate the thresholds that exist in achieving MOMP, thereby predisposing cells to undergo apoptosis, has been the basis of many excellent studies that have either overlapped or converged on the importance of the Bcl-2 homology (BH) -domain containing proteins [4].

Briefly, the Bcl-2 proteins can be broadly categorized as acting in either a pro-apoptotic or anti-apoptotic manner. Whilst these groups act directly in driving or diminishing apoptosis, a third group of proteins, which are functionally and structurally unique, and when over-expressed can sensitize cells to biochemical cues that induce apoptosis, are the BH3-only proteins (or sensitizer proteins). From these three groups, the BAX (pro-apoptotic) and Bcl-2, Bcl-xL or Mcl1 (anti-apoptotic) proteins have gained the most attention over the recent decades, based on their deregulated expression, significance in the development of a number of cancers and their responsiveness to therapeutics [5]. From these, the importance of BAX protein deregulation in cancer development can stem from the loss-of-function genetic frame-shift mutations, which contribute to the prevalence of a number of solid tumors and leukemias [6–8]. Conversely, in the instance of the Bcl-2 and Bcl-xL anti-apoptotic proteins, their genetically deregulated-over-expression can give rise to similar malignancies, such as B-cell lymphoma, prostate cancer, non-small cell lung cancer, Acute lymphoblastic leukaemia and breast cancer [9–12].

As an alternative regulatory mechanism, protein sub-cellular localization and the coordinated manner in which Bcl-2 protein family members can be regulated by factors from the nucleus, lysosome and mitochondria, has also taken on greater significance over the recent years [13]. Synergistically, the structural composition of the Bcl-2 proteins has unveiled how such proteins come to reside at specific subcellular compartments, and whether each member is regulated by other group members of this family through protein-protein interactions (Table 1) [14].

**Table 1:** The Bcl-2 protein family members can be sub-grouped into pro-apoptotic (Pro-), anti-apoptotic (Anti-) and sensitizer (Sen-) members, which originate from distinct genetic loci and encode proteins of varying amino-acid (aa) length and molecular weight (in kilodaltons, kDa). Each member can be localized in the cytoplasm (C), mitochondria (M), endoplasmic reticulum (ER) or the nucleus (N) and can be regulated through its interaction with other protein family members through protein-protein interactions

Protein	Apoptosis	Gene	Size (kDa)	Location	Protein Partners
**BAX**	Pro-	19q13.33	192aa (21.18)	C, M, N, ER	BAK, Bcl-2,Bcl-xL,Mcl1, BID, BIM, NOXA
**BAK**	Pro-	6p21.31	211aa (23.40)	M	BAX, Bcl-2, Bcl-xL, Mcl1, BID
**Bcl-2**	Anti-	18q21.33	239aa (26.00)	C, M, N, ER	BAX, BAK, Bcl-xL, BID, BIM, BAD, PUMA, NOXA
**Bcl-xL**	Anti-	20q11.21	233aa (26.04)	M, C	BAX, BAK, Bcl-2, BID, BIM BAD, PUMA
**Mcl1**	Anti-	1q21.20	350aa (37.33)	C, M, N	BAX, BAK, BID, BIM, PUMA, NOXA
**BID**	Pro-/Sen-	22q11.21	195aa (21.99)	C, M	BAX, BAK, Bcl-2, Bcl-xL, Mcl1
**BIM**	Pro-/Sen-	2q13.00	198aa (22.17)	C, M	BAX, Bcl-2, Bcl-xL, Mcl1,
**BAD**	Pro-/Sen-	11q13.10	168aa (18.39)	C, M	Bcl-2, Bcl-xL
**PUMA**	Pro-/Sen-	19q13.32	193aa (20.53)	M	Bcl-2, Bcl-xL, Mcl1,
**NOXA**	Pro-/Sen-	9q34.30	476aa (50.93)	C, M, N	BAX, Bcl-2, Mcl1,
**SMAC**	Pro-/Sen-	12q24.31	239aa (27.13)	C, M	xIAP1

**Fig. 1:**
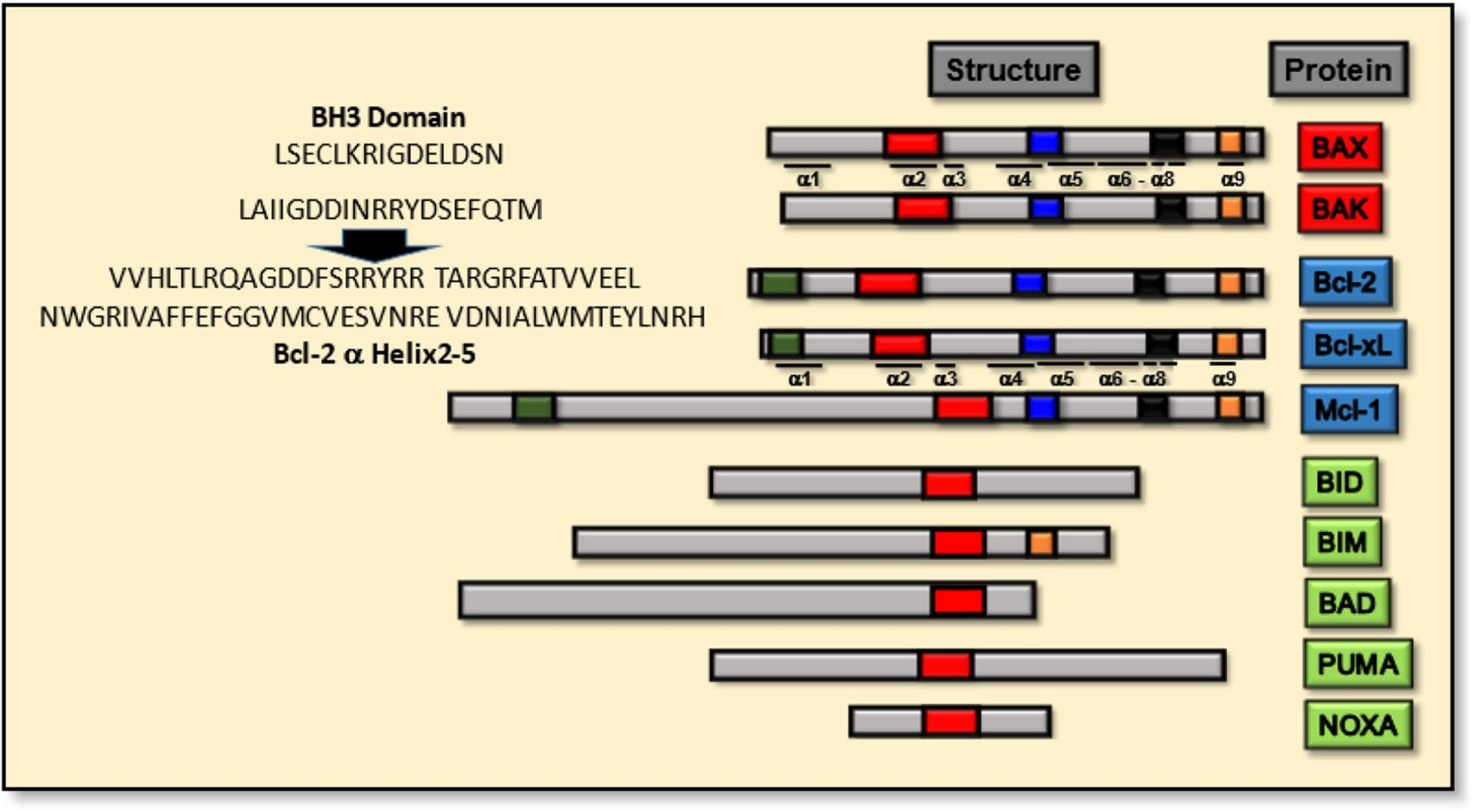
The BH- and helical- domain composition of selected Bcl-2 family pro-apoptotic (red boxes), anti-apoptotic (blue boxes) and BH3-only (green boxes) members. The amino acid sequences of the human BH3-domains from BAX and BAK are highlighted (top left), below which are shown the amino acid sequences of the alpha helices 2–5 from the human Bcl-2 protein. For each of the proteins, the transmembrane domain is highlighted in orange and BH1–4 domains are respectively highlighted in blue, black, red and green for the relevant proteins

In large, the Bcl-2 proteins are composed of conserved BH1-4 domains and in some instances a transmembrane domain (Fig. 1) [4]. Here, the key structural component of intrinsic importance, which is present in all of the pro-apoptotic Bcl-2 family protein members, is unquestionably the BH3 domain, which is a structure composed of ~ 15 amino acids from α-helix 2, and which interacts with the hydrophobic pocket structure formed by α-helices 2-5 of the anti-apoptosis proteins, such as Bcl-2 protein (Fig. 1) [15].

Mechanistically, the interplay between all three groups of proteins, based on their relative abundance in the presence of biochemical activation cues, determine whether the two key intermediates BAX and BAK successfully mediate the formation of a Mitochondrial Pore Complex (MPC), denoted by enlarged mitochondrial cristae and the ultimate release of cytochrome c as a prerequisite to caspases -3, -7 and -9 activation and finally followed by DNA fragmentation [16, 17]. Part of this mechanism incorporates the complex regulatory input from TP53 activation and the key mitochondrial proteins NOXA, PUMA and Smac/DIABLO [18, 19]. As a relatively weak and indirect activator of apoptosis, BH3-*only* NOXA can bind (and inhibit) the *anti*-apoptotic regulators Mcl1 and Bcl-2A1, thus preventing their suppression of BAX- and BAK- protein activation [20–22]. Alternatively, NOXA has also been reported to bind BAX and activate apoptosis in the absence of a BIM, BID and Bcl-xL expression-dependent manner, thereby regulating apoptosis directly [23, 24]. Similarly, BH3-*only* PUMA can induce apoptosis through the direct activation of BAX or BAK [25]. As a negative regulator of apoptosis, Mcl1 has been widely reported as being overexpressed in a number of cancers such as lung and breast [26, 27], and which can be destabilized by NOXA, through the ubiquitination pathway [28, 29]. As an integral regulator of apoptosis, PUMA can also mediate activation of apoptosis through a direct association with Mcl1, that is reversible upon NOXA expression (through it binding Mcl1), thus releasing PUMA by a ‘catch and release’ mechanism [30, 31]. Consequently, from a therapeutic standpoint, Mcl1 expression and regulation have taken on increasing levels of importance, particularly from the contribution they make in offering drug resistance to several cancer therapeutics [32–35]. Like NOXA [36] and PUMA [37, 38], Smac/DIABLO [39] is also resident on the cytoplasmic face of the MOM, and which uniquely allows it to fulfill its role as a positive activator of caspases, through it binding and inhibiting the Inhibitor of Apoptosis Proteins (IAPs), from the extrinsic and intrinsic arms of the apoptosis pathway [40].

**Fig. 2:**
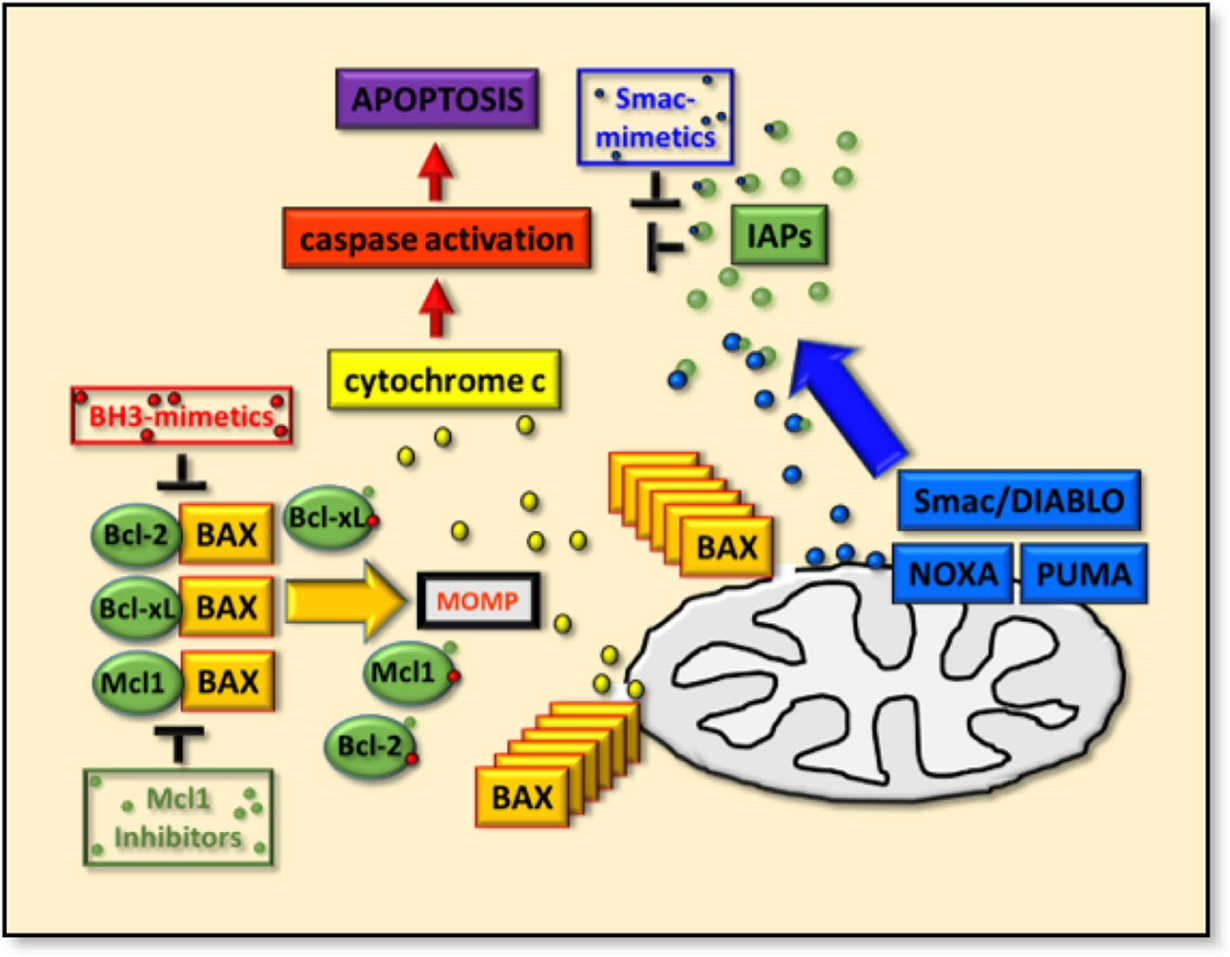
The regulation of apoptosis by the BAX protein through mitochondrial outer membrane permeabilization (MOMP), and the modulation of this key steps by therapeutics. Key negative regulator Bcl-2, Bcl-xL and Mcl1 proteins (solid oval green boxes) for BAX (solid orange box) and their apoptosis inducing effects by cytochrome c release (solid yellow box and circles), caspase protein activation (solid red box) and apoptosis (solid purple box) are shown. The mimetics/inhibitors that can target anti−/pro-apoptotic protein interactions are highlighted as BH3-mimetics (outlined red box, red dots) and Mcl1 inhibitors (outlined green box, green dots), which either induce apoptosis of cells as mono-therapeutics or sensitize them to such effects during combined therapeutic targeting. The blue solid boxes (and small circles) highlight mitochondrial Smac/DIABLO, NOXA and PUMA, which bind the Inhibitor of Apoptosis Proteins (small green circles, IAPs) and the interactions of which can be inhibited by Smac-mimetics (blue outlined box and blue dots)

Collectively, the functional significance of such a small handful of regulatory proteins on cell fate cannot be underplayed, as neither can their mechanistic regulation. While some facets of the latter may be steeped in controversy, many aspects of the BH3-domain containing proteins (and their regulation) have nevertheless been fully harnessed throughout the initial formulation of BH3-mimetics, the emerging Smac-mimetics or the Mcl-inhibitors, and their applications as potential cancer therapeutics, thereafter (Fig. 2).

The principles of BH3-mimetics are mechanistically founded on disrupting the interaction of the pro-apoptotic BH3 domain with the hydrophobic pocket of the anti-apoptotic Bcl-2 proteins (such as Bcl-2, Bcl-xL or Mcl1), thus permitting oligomerized BAX (or BAK) to form the MCP [1]. While this approach is largely dependent on labor-intensive rational therapeutic design strategies, the functional significance of such an approach is rewarded with yielding antagonists that have high efficacy, with demonstrated effectiveness at nano-Molar (nM) concentrations, and which have the potential to be used as either single- or combined- therapeutics [5]. Whilst the founding members of the BH3-mimetic drugs did exhibit multiple protein targets, the subsequent pre-clinical studies that have arisen from such findings have identified a number of additional protein targets. Consequently, when targeted collectively with single therapeutics using combined therapeutic regimens at the pre-clinical and clinical levels, such therapeutics are showing a very high level of effectiveness and generating a growing level of interest in how drugs of this nature can be developed further for greater efficacy.

Synonymously, as progress in therapeutic development has come into fruition, so have the delivery methods that can be utilized for efficient drug delivery to offer maximum effects. Of late, such approaches include the use of self-assembling nanofibers and micelles. The importance of such emerging approaches is highlighted by them offering greater scope in permitting the development and delivery of novel therapeutics that may otherwise be limited by their solubility and availability to cancer cells.

As seen in the context of most cancer-related diseases, the development of BH3-mimetics has been driven by them inducing cell death through apoptosis, a morphologically distinct form of death followed by phagocytosis [41]. Herein, we highlight the importance of this therapeutic paradigm, how it has evolved over time through it being applied in targeting additional key regulatory intermediates from the intrinsic apoptosis pathway. As this has given rise to a number of other potential therapeutics, we describe them in the context of how effective they are as single- or combined-therapeutics in preclinical and clinical models. In doing so, we also address the challenges that have arisen, and how some of them can be addressed through key emerging delivery and targeting approaches, so that novel therapeutics of this nature can be given greater effect for the future.

## Main text

### Basic research and mimetics: a rational drug design strategy

With the contextual origins of BH3-mimetics laying with certain intrinsic pathway intermediate proteins of apoptosis, several approaches have been adopted to develop such therapeutics through specific aspects of rational drug design. For such purposes, the BAX/Bcl-2 regulatory axis has primarily served as a strong foundation to build upon, with particular focus between the 15-amino acid BH3-domain spanning α-helix 2 of BAX [15], and the hydrophobic pocket of Bcl-2 (spanning α-helices 2-5, Fig. 1), for the development of small peptides- and small molecule- inhibitors (SMIs). In furthering such developments, the categorization of genuine mimetics through ‘BH3 profiling’, and whether the potency of candidate agents can induce apoptosis and reduce cell viability in the absence of BAK or BAK expression has also offered good leads in defining therapeutic -authenticity, -specificity and any off-target effects [42–44]. Based on such principles, what has arisen is the diversification of potential antagonists (in both manner and form) and their on-going development and applications in modulating the apoptosis pathway.

**Fig. 3:**
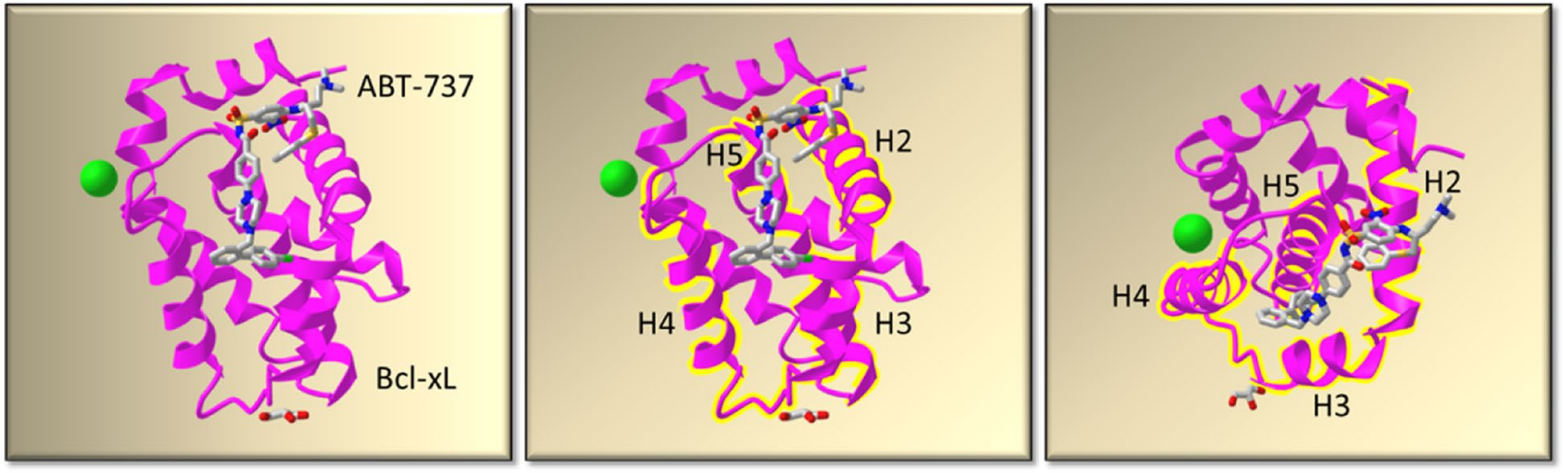
Co-crystal structure of Bcl-xL and small-molecule inhibitor ABT-737. The interaction of alpha-helices (H) 1-9 from Bcl-xL (pink), in combination with ABT-737 (stick diagram) are highlighted in the presence of a chloride ion (green circle) and glycerol (unlabeled lower stick) in the left panel. Bcl-xL α-helices 2-5 (H2-H5) are highlighted in pink and yellow (middle panel) and in the right panel, are shown when viewed from above

Classically, BH3-mimetics have incorporated engineered peptide inhibitors derived from the BH3 domain of the pro-apoptotic activators or sensitizer proteins and designed to bind the hydrophobic groove of their cognate anti-apoptotic proteins [45–47]. Such, unprecedented developments dispelled the belief that such protein-protein targets were ‘undruggable’ at that juncture. While additional criteria of importance included high-affinity binding to targets (within nM ranges) and a dependency on BAK or BAX induced apoptosis [48], some therapeutic peptides were observed to be toxic, unstable and where penetrability was relatively low [49]. However, some candidates emerged to have great potential in acting as apoptosis inducers or sensitizers, either as single agents or in a combined therapeutic approach. Born from such studies were the design and development of small molecule inhibitors, as shown for ABT-737 in Fig. 3 [50].

Through implementing a number of alternative approaches, either exclusively or in a combined manner, through utilizing natural library screening, peptide therapeutics and structure-based design, a new era in therapeutic targeting has arisen, with most of it focusing on the intrinsic arm of the apoptosis pathway for the treatment of hematological or solid cancers [51–57]. As highlighted in Table 2, this field has diversified from the original BH3-mimetics (designed to target the anti-apoptosis regulators), to the development of Mcl1-based inhibitors (to help overcome Mcl1-derived resistance), and beyond towards the development of Smac-mimetics, which more specifically target the Inhibitors of Apoptosis Proteins (IAPs).

**Table 2:** Promising mimetics and inhibitor therapeutic agents for cancer

Agent	Type	Origins	Target	Off-Target	Developer	Ref
**ABT-737**	BH3-M	Structure-based design, BAK peptide	Bcl-2, Bcl-xL	–	Abbott Labs. (IL, USA)	[58]
**Navitoclax**	BH3-M	ABT-737	Bcl-2, Bcl-xL, Mcl1	Mcl1 (weak)	Abbott Labs. (IL, USA)	[59]
**Gossypol** **(AT-101)**	BH3-M	Structure-based design, BIM peptide	Bcl-2, Bcl-xL, Mcl1	–	University of Michigan (MI, US)	[60]
**Obatoclax**	BH3-M	In silico docking studies	Bcl-2, Mcl1	Bcl-xL	University of Montreal (CAN)	[61]
**Venetoclax**	BH3-M	Navitoclax	Bcl-2	Bcl-xL (weak)	AbbieVie (IL, USA)	[62]
**Compound 3**	Smac-M	Smac (AVPI/AVPF peptide sequence)	xIAP, cIAP1/2	NF-κB activation	University of Texas (TX, USA)	[63] [64]
**APG1387**	Smac-M	Smac (AVPI peptide sequence)	xIAP, cIAP1/2	–	University of Michigan (MI, USA)	[65]
**AT-406**	Smac-M	Structure-based design	xIAP, cIAP1/2	–	University of Michigan (MI, USA)	[66]
**Compound A**	Smac-M	Small molecule screen	xIAP, cIAP1/2	–	University of Texas (TX, USA)	[63]
**LC161**	Smac-M	Structure-based design	xIAP, cIAP1/2	–	Dana-Farber CI (MA, US)	[67]
**SM-164**	Smac-M	Structure-based design	xIAP	–	University of Michigan (MI, USA)	[68]
**Birinapant**	Smac-M	Smac (AVPI peptide sequence)	cIAP1	xIAP (weak)	Duke University (NC, USA)	[69]
**A1210477**	Mcl1-I	High throughput screen	Mcl1	–	AbbieVie (IL, USA) Genetech (CA, US)	[70] [71]
**AMG-176**	Mcl1-I	Structure-based design, High throughput screen	Mcl1	Bcl-2, Bcl-xL (minimal)	Amgen (CA, USA)	[72]
**AZD-5991**	Mcl1-I	Structure-based design	Mcl1	–	AstraZeneca (MA, US)	[73]
**S63845**	Mcl1-I	In-silico modelling	Mcl1	–	Institut de Recherches Servier Oncology (FRA)	[74]
**MIM1**	Mcl1-I	Small molecule screen	Mcl1	–	Dana-Farber CI (MA, USA)	[75]
**VU661013**	Mcl1-I	Structure-based design	Mcl1	BIM-Mcl1 destabilization	Vanderbilt University (TN, USA)	[76] [77]
**GDC-0941**	Mcl1-I	In-silico modelling	PI3Kα/δ, Mcl1	–	Piramed Pharma (UK)	[78]

BH3-mimetics (-M), Smac-mimetics (-M) and Mcl1 inhibitors (-I) are highlighted along with the techniques utilized for their discovery or origins. For each drug, we show its cognate target and off-target proteins or effects, its developer and the publication describing its development (Ref)

While some agents show off-target effects, such effects are being harnessed advantageously, through devising novel and combined treatment regimens, based on a common vision shared by the scientific communities from the academic and private sectors. Additionally, most classes of therapeutics exhibit a broad protein target range (with non-specific off targets), whilst some have greater specificity (with minimal off-target effects), as seen for Venetoclax and Gossypol (for BH3-mimetics), and numerous agents for Smac-mimetics and Mcl-inhibitors (Table 2).

Consequently, such efforts have given rise to several promising biological agents, reported to induce the death of several cancer cell types in pre-clinical models, and some of which are currently being driven through clinical trials as very promising anti-cancer therapeutics. In the following sub-sections, we aim to review key aspects of the research efforts published over the last 20 years. As an active area of research that has intensely diversified on a number of fruitful tangents, we aim to initially describe the founding members of the BH3-mimetics group and how the outputs from their evaluation have evolved to give rise to promising therapeutics. We will then turn our attention to how the principles of BH3-mimetics design and targeting have been applied to spawn a number of promising mimetics derived from the NOXA, PUMA and Smac proteins. Lastly in this subsection, we focus on the most encouraging BH3-mimetic (Venetoclax) and the emerging Mcl-1 inhibitors, which are reportingly showing notable pre-clinical outcomes.

### Bcl-2 protein targeting mimetics

Cell fate is determined by the balance of Bcl-2 *anti*-apoptotic protein expression in relation to pro-apoptotic BH3 proteins levels, highlighted by the discovery of Bcl-2 being the first anti-apoptotic overexpressed oncogene, as seen in follicular lymphoma [79–81]. Subsequently, 17–18 member proteins have been reported to share at least one of the four Bcl-2 homology domains (BH1-4), in a family of proteins that exhibit anti- or pro- apoptotic expression-dependent effects [82]. As a protein highly expressed in a number of B-cell lymphomas [83, 84], Bcl-2 is expressed in Chronic lymphocytic leukaemia (CLL) [85], Mantle cell lymphoma (MCL) [85–87], Multiple myeloma (t11;14) (MM) [88] or solid tumors [83], and (in a similar manner to Mcl1 expression) is critical for cell survival [89]. Here, high levels of *pro*-apoptotic protein expression had also been reported [90], thus potentially offering the induction of potent cell death by mono-therapeutic BH3-mimetics acting through disrupting the BH3-domain-hydrophobic groove interaction. Mechanistically, this is permitted through the localization of functionally equivalent BAX or BAK proteins [91] to the outer membrane of the mitochondrion through their α-helix 9 motifs [92–95], where they can homo-oligomerize and mediate the formation of the MPC, thereby inducing the release of cytochrome c and other apoptotic inducing or regulatory factors [96, 97].

### -ABT-737

The BH3-mimetic ABT-737, was designed using an NMR structure-based approach, which targeted the BH3-binding hydrophobic groove of Bcl-2, Bcl-xL (Fig. 3) and Bcl-w (with a Ki of 36 nM), whilst binding minimally to Mcl1 or A1 [50, 98, 99]. Its effectiveness was observed against cell lines and patient samples derived from lymphoma, leukemia and in senescence of solid tumors in single [51, 100] or combined therapy approaches [101–103]. However, resistance against the death-inducing effects of ABT-737 was reported in pre-clinical models, mainly due to upregulated Mcl1 protein expression in certain cancer cell types, and the abrogation of which sensitized cells to death, as seen in normal mouse embryonic fibroblast [99, 104], prostate cancer [34] and breast cancer cells [35]. As a potential mechanism for ABT-737 resistance in pre-clinical models, the significance of such a mechanism in clinical studies remains to be explored in greater detail.

Other aspects of drug resistance were also derived from the stress protein Bcl-2-associated athanogene 3 (BAG3), which could stabilize Mcl1 expression, thus contributing to ABT-737 resistance in breast cancer and prostate cells [105]. Conversely, Mcl1 protein levels were observed to be destabilized when A549 and 95-D cells were treated with Nedaplatin, allowing ABT-737 to induce death with greater efficacy [106], thus highlighting the effectiveness of Mcl1 co-inhibition in a viable combined therapeutic strategy, and which was confirmed through ARC-mediated transcriptional down-regulation of Mcl1 expression [107]. Similar ABT-737-enhancing effects have also been reported upon Mcl1 inhibition in retinoblastoma- [108], melanoma- [109], breast-, prostate-, colon- [110] and liver- cancer cells [111]. While such findings, suggestive of ABT-737 synergizing well with other therapeutics (Table 3) are very encouraging (as seen from its therapeutic activity against a diverse repertoire of cell types), poor bioavailability and low aqueous solubility of ABT-737 did present themselves as major obstacles against its further use in the clinic.

**Table 3:** The single and combined synergizing effects of ABT-737 on cell line viability and/or apoptosis

Cancer	Cell Type	Combined	Reduced Viability	Apoptosis	Ref
**AML**	ex-vivo samples	5-azacytidine	synergized	–	[112]
**Breast**	MDA-MB-231R	irradiation	synergized	–	[113]
**Breast**	T47D	cisplatin	synergized	synergized	[114]
**Breast**	MCF-7, ZR-75-1, MDA-MB231	irradiation	synergized	–	[35]
**Breast**	MDA-MB-435S	VX-680	synergized	–	[115]
**Breast (TN)**	MDA-MB-231	docetaxel	synergized	–	[116]
**CRC**	C26, HCT116, LS174T	oxaliplatin	synergized	–	[117]
**CRC**	HT-29, HCT116	celecoxib	synergized	–	[118]
**Glioma**	LN229, LN18	bortezomib	synergized	–	[119]
**HNSCC**	UM-22A, UM-22B, 1483	cisplatin/etopiside	synergized	–	[120]
**Lung**	A549 and H460 lines	perifosine	synergized	synergized	[121]
**Lung**	A549 and 95-D	nedaplatin	synergized	–	[106]
**Leukaemia**	HL-60, U-937, ML-1, MOLT-4	**-single-**	reduced	–	[122]
**Leukaemia (T-cell)**	MOLT-4	resveratrol	synergized	–	[123]
**Leukaemia**	HL-60	doxorubicin	synergized	–	[124]
**Liver**	HuH-7, HepG2, BEL-7402, SMMC-7721	norcantharidin	synergized	–	[111]
**Liver**	HepG2	curcumin	synergized	–	[125]
**Melanoma**	A375, WM852c	bortezomib	synergized	synergized	[109]
**NSCLC**	A549, H460, H1299, H358, H2009, H1703, H596	cisplatin	**–**	synergized	[126]
**Oral**	MC-3, HSC-3	sorafenib	synergized	–	[127]
**Osteosarcoma**	U-2OS	cisplatin	synergized	–	[128]
**Ovarian/Gastric**	SKOV-3, OVCAR-8, SGC-7901	epothilone B	synergized	–	[129]
**Ovarian**	patient derived organoids	naftopidil	synergized	–	[130]
**Ovarian**	Ovcar-3, Igrov-1	pitavastatin	synergized	–	[131]
**Ovarian**	A2780, cisA2780, IGROV-1, OVCAR, SK-OV-03, primary and xenograft	carboplatin	synergized	synergized	[132, 133]
**Prostate**	DU 145, LNCaP and PC-3	ARC	synergized	–	[107]
**Renal**	PV10, KRC/Y,A498, ACHN	TRAIL	synergized	–	[134]
**Retinoblastoma**	Y79, WERI-Rb	**-single-**	–	–	[108]
**Thyroid**	FTC236, ML1, SW1736, HTh7	doxorubicin/gemcitabine	synergized	–	[135]
**Uterine/Cervical**	SiHa, CaSki	irradiation	synergized	–	[136]
**Breast, Colon, Liver, Pancreatic**	SW480 and LIM1215, Huh-7 and HepG2, HPAC, MDA-MB-231	ARC	synergized	synergized	[137]
**Breast, Colon, Prostate**	MDA-MB-231, HT-29, DU145	methylseleninic acid	synergized	–	[110]

The cancer types are highlighted in bold (left column), the evaluation of single therapy alone is highlighted by ‘-single-‘, non-synergy is highlighted by ‘-‘and the corresponding studies are referenced in the column on the right (Ref). *Abbreviations*: *TN* Triple Negative, *CRC* Colorectal cancer, *HNSCC* Head and Neck Squamous Cell Carcinoma, *NSCLC* Non-small cell lung cancer

### -Navitoclax (ABT-263)

In striving towards overcoming bioavailability and solubility issues for ABT-737, its redesign gave rise to orally administered Navitoclax (ABT-263) [59], which demonstrated a Ki for Bcl-2 of < 0.010 nM and could bind albumin with greater affinity for potential additional delivery benefits in the clinic. While the latter was seen as an encouraging observation, such an interaction was unveiled to reduce the availability of Navitoclax for during the treatment of CLL patients [138]. Regardless of the delivery method of choice, Navitoclax is currently being evaluated and pursued as single- or combined- anti-cancer therapeutic [48], as seen from its ability to sensitize cervical cancer cell lines [139] for example, or synergize with a number of therapeutics for hematological malignancies [140] (Table 4). Despite consistent side effects for Navitoclax, which included thrombocytopenia, based on Bcl-xL expression-dependency for platelet survival, it was effective at killing Bcl-2-dependent CLL cancers, just as effectively as ABT-737 [138].

**Table 4:** The single- and combined- synergizing, or resistance-inducing effects of Navitoclax on cellular apoptosis

Cancer	Cell Type	Combined	Apoptosis	Ref
**AML**	Primary cells	dasatinib	–	[141]
**AML/ALL**	Jurkat, Molt-4	wogonin	synergized	[142]
**Cervical**	SiHa, CaSki	A-1210477	synergized	[139]
**CRC**	HCT116, DLD1, SW48, HT29, HCT-8	apigenin	synergized	[143]
**CRC, Liver**	Huh7, HepG2, BEL7402, HCT116, DLD1, AGS	sorafenib	synergized	[144]
**CRC/Melanoma**	Colo-205	AZD6244 resistance	–	[145]
**Esophageal**	SKGT-4, KATO-TN, YES-6	fluorouracil	synergized	[146]
**Esophageal**	EC109, HKESC-2, CaES-17	**-single-**	–	[147]
**HNSCC**	HN12	fenretinide	synergized	[148]
**Liver**	Huh7	TRAIL resistance	–	[149]
**Lung**	H1650 and H1975	src-inhibitors	synergized	[150]
**Non-small cell lung**	LC2, PC10	cisplatin	synergized	[151]
**Small cell lung**	H209	vorinostat	synergized	[152]
**Lymphoma**	DoHH-2 and SuDHL-4	rapamycin	–	[153]
**Neuroblastoma**	SH-SY5Y and CHLA-119	norcantharidin	synergized	[154]
**Prostate**	LNCaP and PC3	paclitaxel	synergized	[155]
**Prostate**	PC3, C4–2B, C4–2, DU145	MLN2238	synergized	[156]
**Ovarian**	Numerous	paclitaxel/gemcitabine	synergized	[157]
**Liver, Prostate, Cervical, CRC, NSCLC**	Hep3B, PC3, HCT-116, SW480, and SW620, H1299, SK-BR-3, HeLa	metformin	synergized	[158]

The cancer types are highlighted in bold (left column), the evaluation of single therapy alone is highlighted by -single-, non-synergy is highlighted by ‘-‘and the corresponding studies are referenced in the column on the right (Ref). *Abbreviations*: *AML* Acute Myelogenous Leukemia, *ALL* Acute Lymphoblastic Lymphoma, *CRC* Colorectal cancer, *HNSCC* Head and Neck Squamous Cell Carcinoma, *NSCLC* Non-small cell lung cancer

Despite such encouraging outcomes, resistance against Navitoclax was also encountered, due to the induction of Mcl1 [159] or Survivin expression [160], and the direct (or indirect) down-regulation of which, were seen as beneficial to Navitoclax efficacy [142, 143]. For example, inhibition of EGF-R mediated Mcl1 induction improved Navitoclax effects in leukemia K562 cells [161], by destabilizing Mcl1 levels through the upregulation of NOXA expression [148, 154]. Conversely, not all instances of resistance were attributed to Mcl1 expression, as reported for cisplatin-treated non-small cell lung cancer cells (NSCLC), which induced cell death independently of Mcl1 expression levels [151].

### -Gossypol

Other broad-range BH3-mimetics include Gossypol and its derivatives. Derived from cotton seed extracts and identified using NMR binding assays and Fluorescence Polarization displacement assays, racemic Gossypol directly interacted with Bcl-xL and could also counteract the anti-apoptotic effects of Bcl-2, with an IC_50_ of 13.2 μM in MCF-7 cells, as a pan-Bcl-2 inhibitor [52]. Gossypol also inhibits growth and induces apoptosis in several other cell types, such as H1975, H441 and A549 lung cells as a mono-therapeutic in a dose-dependent manner, while also reducing H1975 xenograft growth in mice [162]. It can inhibit EGFR-L858R/T790M signaling, proliferation and migration of NSCLC cells [163], induce death of prostate cancer DU-145 and PC-3 cells [164, 165] and ovarian SKOV3 cancer cells [166]. Fortuitously, racemic Gossypol can also behave as a NOXA-like BH3-mimetic, by selectively promoting apoptosis of cancer cells from the bladder [167], breast [168, 169] and prostate [170], when administered as a mono-therapeutic. Mechanistically, Gossypol can reduce cellular viability upon p53 activation, as seen in LAPC4, PC-3, and DU-145 prostate cancer (PC) cells [171], through ER stress and autophagy in hepatocellular carcinoma (HCC) cells [172], and oxidative stress, as seen in ovarian and MM cells [166, 173]. As a combined therapeutic, it has been reported to induce autophagy and apoptosis in a cell type-dependent manner [174] or exclusively autophagy in melanoma cells [175]. Alternatively, the R(-)-Gossypol enantiomer AT-101, has also been encouragingly reported to reduce invasiveness of rat MLL PC cells [176] and induce mitophagy in U87MG and U343 glioma cells [177]. Mechanistically, Gossypol and AT-101 may contribute to cell death by binding and inhibiting Mcl1, resulting in the sensitization of several cell lines to other therapeutics or through it stabilizing NOXA expression, as in gastric, breast and nasopharyngeal cell lines [178, 179]. Collectively, racemic Gossypol or its enantiomer are showing good potential as *anti*-cancer agents which induce cell death through a variety of mechanisms, and in a variety of cell line-based models. They can be effective as a single- or combined- therapeutics (Table 5), but the activity of which may be limited through the levels of toxicity that arise from administering increasing doses [183].

**Table 5:** The combined synergizing effects of Gossypol and its derivative (AT-101) against certain cancers and cell types

Mimetic	Cancer	Cell Type	Combined	Apoptosis	Ref
**Gossypol**	Glioblastoma	Diff13–20, TS13–20	temozolomide resistance	–	[180]
**Gossypol**	CML	K562	imatinib	–	[181]
**Gossypol**	Colon	HT-29 cells, HCT116, RKO	fluorouracil	–	[182]
**Gossypol**	Nasopharyngeal, Breast, Gastric	MCF-7, YC116, CNE2	gemcitabine	–	[183]
**Gossypol**	Ovarian	OVCAR-3 and MDAH-2774	zoledronic acid	–	[184]
**Gossypol**	Thoracic	H460, TE2, H211	TRAIL	–	[185]
**AT-101**	Bladder	UM-UC2, UM-UC9	gemcitabine, carboplatin	synergized	[167]
**AT-101**	Breast	SKBR-3, MDA-MB-453	trastuzumab	–	[186]
**AT-101**	Pancreatic	BxPC-3	genistein	–	[187]
**AT-101**	Prostate	PC-3 and xenograft	radiation	–	[188]
**AT-101**	Prostate	PC-3 xenograft	docetaxel	synergized	[170]
**AT-101**	Prostate	DU145, PC-3	sorafenib	–	[174]
**AT-101**	Prostate	VCaP	bicalutamide	–	[189]

The therapeutic type is highlighted in bold (left column), non-synergy is highlighted by ‘-‘and the corresponding studies are referenced in the right column (Ref). *Abbreviations*: *CML* Chronic Myelogenous Leukemia

Based on toxicity effects, alternative Gossypol derivatives and analogues have been developed, based on the structural binding properties of the BIM protein BH3-domain with the Bcl-2 protein, namely TW-37 (Compound 5), and which can also bind Bcl-xL and Mcl1 with respective Ki’s of 1110 and 260 nM [60]. As emerging alternatives to Gossypol, their further evaluation in model systems and potential use as single or combined therapeutics are awaited with great eagerness.

### -Obatoclax

Another promising BH3-mimetic, specific for all Bcl-2 proteins includes the rationally-designed and prodiginine-related Obatoclax, which binds the mitochondrially-associated Bcl-2 protein and all pro-survival Bcl-2 proteins with a Ki of 220 nM [61, 190], outlining its suitability for the treatment of hematological malignancies and solid tumors [191, 192]. Good evidence to support this has been derived from xenograft models, where its successful use as a single agent against thyroid cancer, small cell lung cancer and colorectal cancer development has been reported, and efficacy of which can be enhanced when used as a combined therapeutic in limited instances with cisplatin, MEK inhibition, doxorubicin [193, 194], bortezomib, carfilzomib or AZD2281, as highlighted in Table 6.

**Table 6:** The combined synergizing effects of Obatoclax on cellular apoptosis

Cancer	Cell Type	Combined	Apoptosis	Ref
**AML**	U937, HL-60, MV4–11	sorafenib	–	[195]
**Bladder**	HT1197	paclitaxel	–	[196]
**Bladder**	T24, TCCSuP, 5637	cisplatin	–	[197]
**Cholangiocarcinoma**	KMCH, KMBC, TFK,	TRAIL	–	[198]
**Colon**	HCT116, HCT-8,	fluorouracil	–	[199]
**Esophageal**	CaES-17	MG132	–	[200]
**Glioblastoma**	Patient samples	SAHA, LBH589	–	[201]
**Small cell lung**	H82, H526, DMS79, H196, H1963, H69	bortezomib and carfilzomib	synergized	[202]
**Non-small cell Lung**	LoVo, RKO, HCT116	oxaliplatin resistance	–	[203]
**Neuroblastoma**	SK-N-DZ, IGR-NB8	hydroxychloroquine/cisplatin/ doxorubicin	–	[204]
**Pancreas**	BxPC-3	gemcitabine	–	[205]
**Pancreas**	PANC-1 and BxPC-3	TRAIL	–	[206]
**Pancreas**	BxPC-3, HPAC	chloroquine	–	[207]
**Pancreas**	BxPC-3, HPAC, MIAPaCa-2, PANC-1, AsPC-1, CFPAC-1	AZD2281	synergized	[208]
**Thyroid**	KTC-1, BCPAP	LY3009120/vemurafenib resistance	–	[209]

The cancer types are highlighted in bold (left column), non-synergy is highlighted by ‘-‘and the corresponding studies are referenced in the column on the right (Ref). *Abbreviations*: *AML* Acute Myelogenous Leukemia

Moreover, Obatoclax has completed phase I trials for several cancers with encouraging efficacy, but with notable side effects [191, 192].

### NOXA-mimetics

As to be expected NOXA expression plays a relatively restricted role in determining cell fate and tumor progression in the presence and absence of therapeutic treatments against LC [210–213], leukemias [214, 215], rhabdomyosarcoma [216, 217], PC [218], OC [219], colorectal cancer (CRC) [220, 221], melanoma [222] and MM [223]. As a protein up-regulated in CLL, NOXA can interact with Mcl1 and neutralize its anti-apoptotic activity [224], thus offering good justification for the development of NOXA-like BH3-mimetics, and particularly for CLL therapy [225]. Here, Mcl1-derived inhibitors had been reported to be sufficient to induce apoptosis through their specific targeting of NOXA. While encouraging, such an approach may come with limitations, seeing as anti-apoptotic proteins (such as NOXA) are embedded in the mitochondrial membrane and more susceptible to changes in their conformation, and which may consequently respond differently to the affinity of mimetics in comparison to BH3-mimetics which are otherwise directed at soluble targets [14].

### PUMA-mimetics

The BH3-*only* protein PUMA, is frequently down-regulated in a number of tumors [26], and which may contribute to chemoresistance and tumor progression in conjunction with other protein factors, based on the phenotype of PUMA knockout-mice lacking spontaneous oncogenesis [226]. Nevertheless, PUMA-derived helices in the form of SAHBs [227], which have the capacity to bind BAX or Bcl-xL/Mcl1 as dual anti-apoptosis inhibitors that induce BAX activation, have shown some encouraging outputs in overcoming chemoresistance in neuroblastoma cells. As in the instance of BH3-mimetics, the starting point here has been the design of peptide inhibitors, which can potentially take up a helical conformation upon binding the hydrophobic groove of BAX, Bcl-xL or Mcl1 [228, 229], or through them being generated as stapled peptides [228].

### SMAC-mimetics

The human Inhibitor of Apoptosis (IAP) family of proteins are composed of eight members, each of which encode a Baculoviral IAP Repeat domain (BIR) and several of which are over-expressed in hematological malignancies and solid tumors [230–232]. While each member can regulate cell survival in response to a number of signaling cues, cIAPs and XIAPs have direct anti-apoptotic roles [233, 234] and offer chemoresistance when over-expressed in cancer cells [230]. Mechanistically, they can be targeted through homodimers of Smac binding the BIR domain and destabilizing the IAP protein through IAP auto-ubiquitination and degradation [235–238]. Consequently, cells can be sensitized to the apoptosis-inducing effects of TRAIL with PI3K or MAPK inhibition [239, 240] and etoposide or paclitaxel [241] in combined therapeutic approaches. As mono-therapeutics, Smac-mimetics can act through preventing inhibitory IAP binding to caspases -3, -7, -9 [242–245] and thus potentiating the effects of any death-inducing signals [230]. Therefore the loss of Smac expression can negatively impact the full execution of apoptosis, as seen with some cancer patients encoding high levels of Smac expression, and which can be correlated with a better prognosis [230]. Consequently, targeting the Smac-IAP interaction has been explored as a therapeutic strategy through the use of SMIs, peptides [230], LAso Smac-mimetics or Smac anti-sense oligonucleotides, and all of which showed varying degrees of success. For example, Smac-mimetics induced the degradation of cIAPs -1, -2 and -3 *via* the proteasome, leading to NIK activation, NF-κB activation, and up regulation of TNF-α expression and subsequently cell death in an autocrine- or paracrine- manner, in treated fibrosarcoma, colorectal and melanoma cell lines [235, 238]. Generally speaking, Smac-mimetics can be as little as 4 aa long and which can target BIR repeats -3 and -4 of the three cIAPs [246]. While they have shown good effects against specific solid tumors and leukemias as mono-therapeutics [247], they can also sensitize cells to death in combined therapeutic regimens, as seen with temozolomide [248], cytarabine [249], prednisolone [250] and even the pro-inflammatory cytokine TNF-α [251, 252], in a variety of cell types (Table 7).

**Table 7:** The combined synergizing effects of Smac-mimetic (or IAP inhibitor) agents against selected cancers and cell types

Agents	Cancer	Cell Type	Combined	Ref
**APG-1387**	Liver	HepG2 and HCCLM3	TNF-α, TRAIL	[253]
**APG-1387**	Ovarian	SKOV3	TNF-α	[254]
**AT-406**	Osteosarcoma	AT-406, Xenograft	doxorubicin	[255]
**AZ58**	Bladder	UMUC-6, UMUC-12, and UMUC-18	gemcitabine, cisplatin	[256]
**Birinapant**	AML	MLL-ENL AML	emricasan	[257]
**Birinapant**	Breast	SUM190, SUM149	TRAIL	[69]
**Birinapant**	Head and Neck	UM-SCC-46 and -11B xenograft	radiation	[258]
**Birinapant**	Non-small cell Lung	LKB1- and KRAS-mutated	ralimetinib	[259]
**Birinapant**	Ovarian	CAOV3, OVCAR4, SKOV3, OVCAR8, OV90, 1A9	docetaxel	[260]
**Birinapant**	Ovarian	OCAR3, OVCAR8	carboplatin/ paclitaxel	[261]
**BV6**	AML	51% primary AML cells	cytarabine	[262]
**BV6**	Glioblastoma	–	temozolomide	[263]
**BV6**	CRC	SW480, HT-29, HCT-15	radiation	[264]
**BV6**	Glioblastoma	A172, T98G	temozolomide	[265]
**BV6**	Multiple	HT1080, HeLa, Jurkat, L363, MMI, OPM2, RPMI, HT29	TNF-α, TRAIL	[266]
**BV6**	Renal	CaKi1, KTCTL26, 786O, KTCTL30, KTCTL2	interferon-α	[267]
**JP-1201**	CRC	HT-29	radiation	[268]
**JP-1201**	Pancreas	Xenograft MIA PaCa-2	gemcitabine	[269]
**LCL161**	B-Cell Lymphoma	Xenograft Raji/4RH	rituximab, gemcitabine, vinorelbine	[270]
**LCL161**	Breast	MCF7-TamC3	tamoxifen	[271]
**LCL 161**	HNSCC	human cell culture, xenograft	radiation	[272]
**LCL161**	HNSCC	PCI-1, PCI-9, PCI-13, PCI-52, PCI-68	FAS-L	[273]
**SM-164**	Breast	(SK-BR3) and (MDA-MB-468	radiation	[274]
**SM-164**	Breast, Prostate, Colon	Cell lines	TRAIL	[275]
**SM-164**	Pancreatic	Panc-1, AsPC-1, BxPC-3	gemcitabine	[276]
**compound A**	Bladder	UC-9. UC-14, RT4 v1, RT4 v6	TRAIL	[277]
**compound 3**	Pancreas/CRC	Panc-1 and HCT116	doxorubicin	[278]
**Debio 1143**	Lung	LLC-OVA	radiotherapy	[279]
**SH122**	Prostate	DU145, CL1	TRAIL	[280]
**SW IV-134**	Pancreatic	PANC-1, CFPAC-1, BxPC-3, AsPC-1, MIA PaCa-2	gemcitabine	[281]

The therapeutics types (Agents) are highlighted in bold (left column) and the corresponding studies are referenced in the column on the right (Ref). *Abbreviations*: *AML* Acute Myelogenous Lymphoma, *CRC* Colorectal cancer, *HNSCC* Head and Neck Squamous Cell Carcinoma

Smac-mimetics or IAP inhibitors have also shown effectiveness with death receptor agonists in a variety of cell types, as seen against TRAIL in -CLL [282], -ALL [283] and -cholangiocarcinoma cell types [284], or with radiation-induced death of glioblastoma cells [285]. Similarly, signal transduction inhibitors have been reported to improve efficacy of treatments when administered in combination with Smac-mimetics, as seen with 5-Aza against AML cells [286, 287], tyrosine kinase inhibitors against leukemia cells [67, 288], CD95 agonist antibodies against leukemia cells [289], or Smac-mimetics and TNF-α against alveolar epithelial cells [290]. Lastly, some Smac-mimetics exhibit synergistic effects with Birinapant-induced death of liver cancer cells [291], triple-negative BC cells (TNBC) [292], ovarian cancer (OC) cells [260] and primary AML cells in response to BV6 [262].

### -Venetoclax (ABT-199)

Venetoclax was developed through rational design approaches as a high-affinity antagonist for Bcl-2 (with a Ki < 0.010 nM) and a 4000 fold lower affinity binding for Bcl-xL, to help overcome thrombocytopenia side effects derived from off-target Bcl-xL inhibition, and a common feature associated with ABT-737 and Navitoclax treatments [62]. While Venetoclax could induce therapeutic resistance by up-regulating Bcl-xL and Mcl1 expression in some instances [293], it has shown encouraging results as a single agent for treating acute lymphocytic leukemia (ALL) [294], head and neck squamous cell carcinoma (HNSCC) [295] and neuroblastoma [296, 297] in pre-clinical models. Through structure-based design, other ABT-737-derived SMIs targeting Bcl-2 and Bcl-xL, have also arisen in the form of BM-957 and BM-1197, which showed improved solubility, pharmokinetic properties and tumor regression capabilities [298, 299], as single therapeutics against AML [300]. More specifically, such agents spared platelets [62] and to avoid therapeutic resistance, Gemcitabine was reported to effectively decrease Mcl1 expression, while enhanced Bcl-2 expression (in pancreatic cancer cells), could be efficiently targeted by Venetoclax through it beneficially enhancing expression of BIM [301]. Similarly, Venetoclax has shown encouraging results for synergizing with a growing array of therapeutics in pre-clinical models for several cancer cell types, as a well-tolerated combined therapeutic (Table 8).

**Table 8:** The combined synergizing effects of Venetoclax on cellular apoptosis

Cancer	Cell Type	Combined	Apoptosis	Ref
**ALL**	LOUCY cell line	doxorubicin, l-asparaginase, and dexamethasone	–	[302]
**AML**	Primary cells and U937	daunorubicin or cytarabine	–	[303]
**AML**	KOPT-K1	S63845	synergized	[304]
**AML**	MV4–11 and MOLM-13, KG-1a, U937, and THP-1	triptolide	synergized	[305]
**AML**	Jurkat and Molt4	gemcitabine	synergized	[306]
**AML**	Molm14 and OCI-AML3	VS-4718	–	[307]
**AML/MDS/CMML**	Ex-vivo samples	5-azacytidine	–	[112]
**Breast**	23 T Xenografts and MCF7	tamoxifen, AZD8055	–	[308]
**CML**	KCL22	imatinib	sensitized	[309]
**CRC**	Xenograft and RKO cell line	LZT-106	synergized	[310]
**Diffuse BCL and FL**	Cell lines and TMD8 xenograft model	ibrutinib	synergized	[311]
**Leukemia, Lymphoma**	SU-DHL-4, OCI-Ly1 199R, SC-1199R and BCl and FL primary samples	A-1592668 and analogue A-1467729	synergized	[312]
**Nasopharyngeal**	CNE-2, 5-8F	S63845	synergized	[293]
**MM**	OPM2, H929	THZ1	synergized	[313]
**MM**	U266, KMS11, OPM2, RPMI8226 and KMS28-PE	flavopiridol	synergized	[314]
**Pancreatic**	MIA Paca-2 xenograft	gemcitabine	–	[301]
**Soft Tissue Sarcoma**	Rhabdomyosarcoma, SW982 (synovial sarcoma) cells or primary cells	bortezomib	synergized	[315]

The cancer types are highlighted in bold (left column), non-synergy is highlighted by ‘-‘and the corresponding studies are referenced in the column on the right (Ref). *Abbreviations*: *AML* Acute Myelogenous Leukemia, *ALL* Acute Lymphoblastic Lymphoma, *MDS* Myelodysplastic syndrome, *CMML* Chronic myelomonocytic leukemia, *BCL* B-cell lymphoma, *FL* Follicular Lymphoma, *MM* Multiple myeloma

### Mcl1-inhibitors

As the first homologue of Bcl-2 found to be overexpressed in a number of hematological malignancies such as MM [316], and which conferred anti-apoptotic effects under normal conditions [317–320], Mcl1 expression was also reported to contribute to chemoresistance, thus highlighting its suitability to be targeted in combined therapeutic approaches. Normally, Mcl1 is localized to the MOM [321], ER, nucleus [322, 323], and mitochondrial matrix [321] in conjunction with its spliced variants Mcl-1S [324–326] and Mcl-1ES [327]. As a protein that is essential for survival of a number of normal cell types [328] it has been reported to be over-expressed in a number of other cancers, such as B- and T- non-Hodgkin’s lymphoma [329], and solid tumors, such as hepatocellular carcinoma (HCC) [330], esophageal squamous cell carcinoma (SCC) [331] and breast cancer (BC) [332].

Based on these properties of Mcl1 expression, Mcl1-inhibitors have been eagerly pursued, giving rise to the discovery of Prodigiosin for example, which is a natural compound that targets the hydrophobic groove of Mcl1 with good specificity [333]. Based on the normal role of Mcl1 for hematopoietic stem cell survival [334] and oxidative phosphorylation [321], the development of most Mcl1 antagonists has been seen to offer potential limitations when used as a mono-therapeutic.

Alternative Mcl1-specific mimetics or inhibitors are also showing their usefulness as mono- or combined- therapeutics, as seen with S63845-mediated apoptosis of MM and NSCLC, gastric cancer (GC), PC, [74] and T-cell acute lymphoblastic leukaemia (T-ALL) cells [304] or ABT-199 and S63845 co-treatments in cervical cancer cells [335]. Other, promising Mcl1-specific candidates as combined therapeutics include, A-1210477 (Ki, 0.45 nM) [70], AZD5991 (Ki, 0.13 nM) [73] and AMG-176 (Ki, 0.13 nM) [72], AM8621 (Ki, 0.06 nM) [72], S64315 (Ki, 0.048 nM) [336] and VU661013 (Ki, 97 pM) [76], as outlined in Table 9.

**Table 9:** The combined synergizing effects of Mcl1-inhibitors against selected cancer and cell types

Agents	Cancer	Cell Type	Combined	Ref
**A1210477**	AML	THP-1 U937	venetoclax	[337]
**A1210477**	Breast	MDA-MB-231 cells	TRAIL	[338]
**A1210477**	Cervical	SiHa and CaSki	navitoclax	[139]
**A1210477**	CML	K562, K562/R	EE-84	[339]
**A1210477**	CRC	RKO, HT29, A375	cobimetinib	[340]
**A1210477**	DLBCL	U-2946	navitoclax	[341]
**A1210477**	PC, GC, NSCLC, MM	BxPC-3, EJ-1, H23, and OPM-2	navitoclax	[70]
**A1210477**	HNSCC	PCI15B, Detroit 562, MDA686LN, and HN30	navitoclax	[342]
**A1210477**	nHL	SU-DHL-4, WSU-NHL, WSU-DLCL2, KARPAS-422	venetoclax	[343]
**AMG-176**	CLL	Patient samples (5)	venetoclax	[344]
**AZD-5991**	AML	OCI-AML3 and MCL-1-OE Molm13 and MV4–11	venetoclax	[345]
**AZD5991**	MM	NCI-H929	bortezomib, venetoclax	[73]
**GDC-0941**	Breast	MDA-MB-231, SKBR3	ABT-737	[346]
**Mim1**	Glioblastoma	U87mg	temozolomide	[347]
**Mim1**	Melanoma	C32 melanoma cells	dacarbazine	[348]
**Mim1**	MM	Colo829	dacarbazine	[349]
**S63845**	AML	OCI-AML3, MOLM-13, OCI-AML2	trametinib/HDM201	[350]
**S63845**	AML	Primary samples	venetoclax	[351]
**S63845**	AML	MOML-13, SKM-1,	trametinib	[352]
**S63845**	AML	Cell lines and primary cells	venetoclax	[353]
**S63845**	Breast	SK-BR-3	docetaxel, trastuzumab, lapatinib	[354]
**S63845**	CRC	HCT116	regorafenib	[355]
**S63845**	Mantle cell lymphoma	Patient-derived xenografts	venetoclax	[356]
**S63845**	Melanoma	Patient samples	navitoclax	[357]
**S63845**	Melanoma	MeWo	TRAIL resistance	[358]
**S63845**	Myeloma	U266 xenograft	venetoclax	[359]
**S63845**	MM	MOL-P8, OPM-2, NCI-H929	venetoclax, bortezomib	[360]
**S63845**	MM	RPMI-8226 xenograft	venetoclax	[361]
**S63845**	Nasopharyngeal carcinoma	CNE-2, 5-8F	venetoclax	[293]
**S63845**	T-ALL	Zebrafish T-ALL cells	venetoclax	[304]
**VU661013**	AML	MV-4-11, AML-001/2, patient xenografts	venetoclax	[76]
**VU661013**	Breast	HCC1428, MCF7, T47D	navitoclax	[362]

The therapeutic types (Agents) are highlighted in bold (left column) and their corresponding studies referenced in the column on the right (Ref). *Abbreviations*: *CML* Chronic Myelogenous Leukemia, *CRC* Colorectal cancer, *DLBCL* Diffuse large B-Cell Lymphoma, *PC* Prostate Cancer, *GC* Gastric Cancer, *NSCLC* Non-small cell lung cancer, *MM* Multiple myeloma, *HNSCC* Head and Neck Squamous Cell Carcinoma, *nHL* Non-Hodgkin’s Lymphoma, *CLL* Chronic lymphocytic leukemia, *AML* Acute Myelogenous Lymphoma, *T-ALL* T-cell acute lymphoblastic leukemia

From this group, AZD5991 showed promising effects [363, 364], either as a monotherapy or as a combined agent [73], to treat a number of hematologic and solid tumor cell lines. It also showed combined effectiveness against biomarker-specific and Venetoclax-resistant AML cells, highlighting its usefulness for cell line- or biomarker- specific cancers [365]. Of similar interest is AZD0466, which can also be administered using nanoparticle technology as novel delivery method [366]. Alternatively, S63845 [74] is also showing good promise, although its optimal use is not fully

defined [367], but nevertheless, can be used as a monotherapy against T-ALL [304] using MOLT-3, RPMI-8402 or neuroblastoma patient cells [297] and can be delivered using nanoparticles to improve remission and therapeutic indices [368].

As drug resistance can be mediated by Mcl1 up-regulation in a number of cancer types [369], which can be sensitized to death upon Mcl1 down-regulation (as in the instance of some ABT-737 co-treatments) [99], Mcl1 inhibitors are therefore viewed as having better potential for combined chemotherapeutic treatments. Alternative approaches that induce Mcl1 proteasomal degradation (by GDC-094 treatments, for example), have also been effective in overcoming resistance, thus allowing ABT-737 to have greater effects against BC cells [346] and lung cancer (LC) cells [106]. Similar effects have also been reported for Mcl1 inhibition and TRAIL co-stimulation of BC cells [338]. Conversely, stabilization of Mcl1 protein by BAG3 and survival of BC and PC cells under ABT-737 stimulatory conditions has also been reported [105], highlighting a central but indirect and positive regulatory mechanism for the Mcl1 protein as a therapeutic resistance factor. In a similar manner, the interaction of Mcl1 with other protein regulators cannot go ignored and may help in overcoming ABT-737 resistance, as seen from the NOXA protein interacting with Mcl1 [211, 370]. Here, enhancing NOXA levels, suppressed the anti-apoptotic actions of Mcl1 and override resistance to ABT-737, with Vorinostat [152], Vinblastine [371], Bortezomib [109], Dinaciclib [372] co-treatments in SCLC, CLL and melanoma cells.

Transcriptional repression of Mcl1 expression has also been reported as an effective approach to overcoming drug resistance as reported in the instance of HCC sensitization to ABT-737 and Norcantharidin co-treatments [111]. Alternatively, inhibition of Mcl1 can also be induced through mitochondrial stress or under Obatoclax stimulatory conditions, which can initiate autophagy-dependent necroptosis as an alternative to cell death by apoptosis [373].

As mentioned, of emerging interest are studies being performed in the presence of specific genetic biomarkers [351]. For example, AML primary samples with an IDH2–140 mutation were more sensitive to Venetoclax as a single agent, whereas samples with a FLT3-ITD mutation were more resistant, which is an effect that could be reversed with S63845 co-treatments [351]. Similarly, AMG-176 [72] has also been shown to be effective as a monotherapy directed against Mcl1 in CLL lymphocyte patient samples [344] and in combination with Venetoclax [344]. Lastly, stapled peptides can also activate and promote BAX/BAK/BIM proteins [374], which also validates findings from previously published structural studies [375, 376]. For example Bim_S_2A, a hydrocarbon stapled BIM BH3-peptide can override Mcl1 mediated drug resistance in cell lines [377] only when Bcl-xL is absent or neutralized [378]. Additionally, Mcl1-specific MIM1 was identified in a stapled peptide-based screen [75], which showed good efficacy as a monotherapy against colo-829 melanoma cells, and which synergized the death inducing effects of ABT-737 or Decarbazine [349, 379]. When taken with reports that the Mcl1 hydrophobic groove is more rigid than the hydrophobic groove of Bcl-xL [14], targeting Mcl1 may be permitted with greater specificity and affinity and which may even offer some very promising outcomes with minimal non-specific side effects towards Bcl-xL. Consequently, induced Mcl1 expression can potently enhance drug resistance in cancer cells treated with BH3-mimetics and Mcl1 inhibitors are showing their true potential in over-coming this when utilized in combined- therapeutic treatments, in pre-clinical studies.

To summarize, a growing number of BH3-mimetics have been designed and show good efficacy for inducing cell death at nM quantities. Here, compounds such as ABT-737, Obatoclax and Gossypol, have even been reported to show broader specificity by displacing *anti*-apoptotic proteins Bcl-2, Bcl-xL and Mcl1, thus enhancing their effectiveness at inducing or sensitizing cells to apoptosis in a number of hematological cancers and solid tumors [51, 61, 100, 190, 380–382]. However, such studies have also indirectly unveiled the importance of Bcl-xL in platelet homeostasis, thus inspiring alternative targeting strategies, and which arrived at the further development of Navitoclax- and Venetoclax- derivatives, which eliminate Bcl-xL protein levels through protein degradation [383, 384]. Limitations to one side, such stoic examples offer great promise as therapeutics, the development of which have also encouraged avenues of research to overcome important considerations, such as off-target effects, bioavailability, and solubility.

In view of the surge in efforts at developing promising mimetics at the preclinical level, one central question that has been raised originates from how well basic-research efforts have developed in the direction of translational medicine. Although the Venetoclax and Mcl1-inhibitor paradigms lay strong foundations for other mimetics to developmentally follow suit by (at the pre-clinical and clinical level), a significant number of the resulting therapeutics still remain to be evaluated in the clinic. Therefore, in the following sections we highlight what progress has been made over the last 5–10 years in bringing the most promising aspects of specific therapeutic strategies towards fruition, as seen from published clinical trials.

### BH3-mimetics and Bcl-2 protein inhibition: a clinical perspective

#### -ABT-737

Although ABT-737 showed promising therapeutic properties in pre-clinical cell line-, primary cell- and animal-models against cancers over-expressing Bcl-2 or Bcl-xL [51], to date only two clinical trials for ABT-737 have been publicized (www.clinicaltrials.gov). These addressed the effects of platinum combined with ABT-737 against ovarian cancer (NCT01440504) and the use of ABT-737 ex-vivo on apoptosis of platelets in idiopathic thrombocytopenic purpura patients co-treated with Eltrombopag and Romiplostim (NCT00902018, [385]). While the findings from the former study remain to be published, in the latter study (NCT00902018), 8 h and 3 h treatments of ABT-737 sensitized patient platelets to apoptosis, and Eltrombopag pre-treatment for 1 week gave rise to therapeutic resistance towards ABT-737, possibly due to a recorded increase in the ratio of Bcl-xL:BAX proteins or enhanced Akt signaling and Mcl1 expression in patient samples. As the prototypical BH3-mimetic [51], ABT-737 did encouragingly enter clinical trial phases I/II, but poor solubility and oral bioavailability [59, 386] did offer limitations in its dose adjustments during combined or single treatment approaches [387].

#### -Navitoclax (ABT-263)

As the orally available analogue of ABT-737, promising findings from phase I/II clinical trials with Navitoclax have been recorded for its safe tolerance [388] against various types of cancer [389–391]. To date, 34 clinical trial studies have been registered (www.cliniclatrials.gov), of which 17 have been completed and 12 published in depth (Table 10).

**Table 10:** Selected phase I clinical trials conducted with Navitoclax as a single or combined therapeutic in untreated and pre-treated patients with Docetaxel (DOC), Erlotinib (ERLO), Gemcitabine (GEM), Carboplatin (CARB), Paclitaxel (PAC), Etopiside (ETOP), Cisplatin (CISP) against Advanced Solid Tumors (AST), non-small cell lung cancer (NSCLC), Prostate Cancer (PC), Squamous cell carcinoma (SCC) and lymphoid malignancies (LM) for Maximum Tolerated Doses (MTD) outcomes

Navitoclax	Combined	Patients	Disease	Outcomes	ORR	Stabilized	Ref
**NCT00888108**	DOC	39/41 Pre-Treated	AST	MTD	4/35 PR	–	[392]
**NCT01009073**	ERLO	–	NSCLC, PC, SCC	MTD	0% ORR	27%	[393]
**NCT00887757**	GEM	Pre-Treated	ST	MTD	0% ORR	54%	[394]
**NCT00891605**	CARB/PAC	–	ST	TERMINATED	5.3% PR	36.80%	[395]
**NCT00878449**	ETOP/CISP	Untreated (14 days)	SCLC	MTD	–	–	[396]
**NCT00445198**	**-single-**	Pre-Treated	NSCLC, ST	0%	1/47 PR	22.8% (13 m)	[390]
**NCT00406809**	**-single-**	–	LM	MTD	10/46 PR	–	[389, 397]

The clinical trials reference numbers highlighted in bold (left column), the evaluation of a single therapy alone is highlighted by -single-. Objective Response Rates (ORR) and Partial Responses (PR) are expressed as responding patient numbers/numbers assessed, or as percentages (%). Disease stabilization (Stabilized) effects (as percentage responders) are highlighted in months (m). The corresponding references for the studies are highlighted in the column on the right (Ref)

As a single agent, it has been tested with CLL (NCT01557777), platinum resistant ovarian cancer (NCT02591095) and in combination with other drugs, against hematological and solid cancers [192] such as Rifampicin (NCT01121133, [398]), and Ketoconazole (NCT01021358). Results for some of these trials are eagerly awaited. Among the hematological neoplasms, Navitoclax was evaluated in combination with Rituximab in phases I/II clinical trial studies, conducted in patients with relapsed or refractory CD20+ lymphoid malignancies and patients with B-cell CLL with no prior treatment (NCT01087151), respectively. Here, combinations of Navitoclax and Rituximab were tolerated and showed significant synergistic effects in both settings [399, 400]. Additionally, Navitoclax showed encouraging disease stabilization properties as a single therapeutic or in combination with Gemcitabine in NSCLC and solid tumor patients, although side effects remained as on-going concerns. In this context, and more recently among hematological cancer patients, Venetoclax was evaluated in combination with Navitoclax (NCT03181126) to determine safety and pharmokinetic properties in a phase I trial for relapsed or refractory ALL or LL patients and which offered more encouraging outcomes, highlighting promising potential for BH3 mimetics to be used in combination with each other in addition with pre-existing therapeutics.

#### -Gossypol

AT-101, the orally available enantiomer of racemic Gossypol, showed acceptable *anti*-cancer properties in a range of models (Table 11), and which led to it being evaluated further in clinical trials.

**Table 11:** Selected phase I-II clinical trials conducted with AT-101 as a single (**−s**ingle-) or combined therapeutic on untreated/pre-treated patients with Carboplatin (CARB), Paclitaxel (PAC), Cisplatin (CIS), Etoposide (ETOP), Luteinizing Hormone Receptor Hormone (LHRH) agonist, Bicalutamide (BIC) against Advanced Solid Tumors (AST), Giant Cell Glioblastoma (GCG), Adrenocortical (ADC), Solid Tumors (ST), Small Cell lung Cancer (SCLC) and Metastatic Prostate Cancer (MPC) are highlighted for Maximum Tolerated Doses (MTD). Side effects are abbreviated as ADP (Abdominal Pain), Neut (Neutropenia), Throm (Thrombocytopenia), Gastrointestinal symptoms (GI), Fatigue (FAT), Anemia (Anem) and Nausea (Nau). Objective Response Rates (ORR), Complete Responses (CR), Partial Responses (PR), Prostate-Specific Antigen levels (PSA) and percentage patients (%) from the whole group experiencing disease stabilization effects are also highlighted (Stab**.**). The clinical trials reference numbers are highlighted in bold (left column), and the corresponding references highlighted in the column on the right (Ref). Unavailable data is highlighted by ‘-‘

AT-101	Phase	Patients	Patients	Combined	Disease	Adverse Effects	ORR	Stab.	Ref
**NCT00891072**	I	24	Pre-treated	CARB/PAC	AST	ADP/Neut/Throm	4.16% CR; 16.66% PR	33%	[401]
**NCT00540722**	II	56	Untreated (3 wks)	**-single-**	GCG	GI/FAT	–	–	–
**NCT00848016**	II	29	–	**-single-**	ADC	Anem/Naus/FAT	–	–	–
**NCT00544596**	I	27	Untreated (4 wks)	CIS/ETOP	ST/SCLC	–	–	–	–
**NCT00773955**	II	14	Pre-treated	**-single-**	SCLC	Anem/GI	0% CR; 0% PR	–	–
**NCT00666666**	II	55	Untreated (4 wks)	LHRH/BIC	MPC	Anem/GI/AT	18–60% Decreased PSA	–	–

To date, 29 clinical trial studies have been registered (www.clinicaltrials.gov), 17 completed and 5 of which have been published in detail (Table 11). As a mono-therapeutic, it has been tested in several phase II trials addressing its efficacy in NSCLC (NCT00773955), adrenocortical cancer (NCT00848016) and giant cell glioblastoma (NCT00540722) with significant adverse gastrointestinal effects (such as diarrhoea and nausea) or thrombocytopenia and neutropenia, with little significant benefits. Nevertheless, testing has advanced to determine efficacy in relation to genetic biomarker expression, such as the Bcl-2 family of BH3-proteins and the results for which are eagerly awaited (NCT00540722). AT-101 has also been tested in combined therapeutic approaches in trials for solid tumors and hematological cancers such as SCLC (NCT00397293, NCT00544596), NSCLC (NCT00544960), CLL (NCT00286780), relapsed or refractory SCLC (NCT00397293) and with androgen ablation therapy in PC (NCT00666666). While some of the studies are yet to report their findings, AT-101 with androgen ablation did encouragingly reduce circulating levels of Prostate-Specific Antigen (PSA) in PC patients (NCT00666666). Based on AT-101 binding Mcl1, further work in this area may address how this agent synergizes with other well-established (or promising) BH3-mimetics, or in overcoming enhanced Mcl1-expression mediated Venetoclax resistance.

#### -Obatoclax

As an antagonist that binds the BH3-domain of apoptotic proteins, Obatoclax has been reported to induce cell death, cell arrest and autophagy in leukemia and lymphoma cell lines [190, 402–407]. To date, 20 clinical trials for Obatoclax have been registered (www.clinicaltrials.gov), of which 12 have been completed, and 8 of which have been described in detail (Table 12).

**Table 12:** Selected phase I-II clinical trials conducted with Obatoclax as a single (−single-) or combined therapeutic in untreated/pre-treated or non-refractory(−)/refractory (Refrac) patients with Carboplatin (CARB), Etoposide (ETOP) or Topotecan (TOPOT) against extensive-stage small cell lung cancer (es-SCLC), Myelodysplastic Syndrome (MDS), Hodgkin’s Lymphoma (HL), Myelofibrosis (MFS), advanced Chronic Lymphocytic Leukemia (a-CLL), and Hematologic Malignancies (HM). Adverse effects are abbreviated as Neut (Neutropenia), Anem (Anemia), Euph (Euphoria), Dizz (Dizziness), Naus (Nausea), Atax (Ataxia) and Throm (Thrombocytopenia). Disease stabilization effects on patient numbers (expressed as a percentage (%) of the whole group or as positive responders/group size) are highlighted in weeks (>wks). Unavailable data for disease stabilization effects is highlighted by ‘-‘. The clinical trials reference numbers highlighted in bold (left column) and their corresponding references highlighted in the columns on the right (Ref)

Obatoclax	Phase	Patients	Refrac	Combined	Disease	Adverse Effects	Stabilization	Ref
**NCT00684918**	I/II	Untreated	–	**-single-**	AML	Neut	4/19 for 11 cycles	[408]
**NCT00682981**	II	Untreated	–	CARB/ETOP	es-SCLC	Neut/Anem	–	[409]
**NCT00413114**	II	Untreated	–	**-single-**	MDS	Euph/Naus	50% (> 12 wks)	[410]
**NCT00359892**	II	–	Yes	**-single-**	HL	Euph/Dizz	38% (> 8 wks)	[411]
**NCT00521144**	II	Pre-treated	Yes	TOPOT	SCLC	Throm/Neut/Anem/Atax	56% (Phase II)	[412]
**NCT00360035**	II	Pre-treated	–	**-single-**	MFS	Atax/Anem/Throm	–	[413]
**NCT00600964**	I/II	Pre-treated	Yes (22/26)	**-single-**	a-CLL	Atax/Euph/Anem/ Throm	–	[414]
**NCT00438178**	I	N/A	Yes	**-single-**	HM	Neut/Anem/Throm	–	[415]

Obatoclax has been tested in a number of phase I/II trials against SCLC (NCT00682981, NCT00521144), NSCLC (NCT00405951), Hodgkin’s Lymphoma (NCT00359892), MCL (NCT00407303), CLL (NCT00600964), and AML (NCT00684918), with marginally-encouraging outcomes as a single agent or in a limited number of combined studies with common side effects. This may be attributed to it beneficially targeting Bcl-xL (in addition to Bcl-2 and Mcl1) to differing degrees (at a fixed dose), albeit with limited clinical benefits (such as disease stabilization) as a dual-acting drug. Alternatively, in utilizing a combined therapeutic approach, mono-therapeutics (exclusively specific for Bcl-2, Bcl-xL or Mcl1), are being seen to have the advantage of being optimized in doses, to target each of these components more accurately based on the degree of resistance encountered, and may (in some respects) be a more fruitful approach [191, 192, 410, 416, 417]. Based on the benefits arising from Obatoclax stabilizing the progression of certain cancers as a mono-therapeutic, there does therefore exist some potential in how it may be optimized further for this purpose, and possibly thereafter in combined therapeutic regimens.

### SMAC-mimetics

As an important emerging therapeutic group with great potential based on pre-clinical studies, 10 clinical studies for the Smac-mimetic LCL-161 have been registered to date (www.clinicaltrials.gov), of which 6 have been completed and 3 of which have been described in detail (Table 13).

**Table 13:** Selected phase I-II clinical trials conducted with LC-161 as a single (−single-) or combined therapeutic in pre-treated or refractory (Refrac) patients with Paclitaxel (PAC), against Advanced Solid Tumor (AST) diseases (Dis.) and Outcomes for Maximum Tolerated Doses (MTD) are highlighted. Adverse effects (Adv. Effects) are highlighted as Neut (Neutropenia), Gastrointestinal symptoms (GI), Diarrhoea (Diar), Nausea (Nau), Vomiting (Vom) and Anemia (Anem). Objective Response Rates (ORR), Partial Responses (PR), Progressive Disease (PD) and disease stabilization effects (Stabil.) are highlighted as percentage (%) positive-responders. The clinical trials reference numbers highlighted in bold (left column) and their corresponding references highlighted in the columns on the right (URL/Ref). Biomarker assessments (Bio.M) are highlighted and unavailable data is highlighted by ‘-‘

LCL-161	Phase	Patient	Refrac	Combined	Dis.	Outcomes	Adv. Effects	ORR	Stabil.	Bio.M	URL/Ref
**NCT01240655**	Ib	Pre-treated	Yes	PAC	AST	MTD	Neut/GI	27.6% PR; 25% PD	36.8%	–	www.novctrd.com
**NCT01968915**	I/II	–	–	**-single−**/ PAC	AST	–	Neut/Diar	DISC.	–	–	www.novctrd.com
**NCT01098838**	I/II	Pre-treated	–	**-single-**	AST	MTD	Naus/Vom/ Anem	0% ORR	19%	cIAP	[418]

Additionally, phase I studies of TL32711, LCL-161 and HGS1029 Smac-mimetics have been conducted (orally and intravenously), in patients with solid tumors and lymphoma, revealing mixed outcomes ranging from being well-tolerated to inducing cytokine release syndrome. As expected, the latter can be explained through clinical biomarker studies showing the degradation of cIAP and up-regulation of NF-κB activation and *pro*-inflammatory cytokine expression [418–421]. Further optimization of such approaches may help overcome such side effects. Nevertheless, LCL-161 does exhibit disease stabilization (Table 13), in relation to other initial phase I trials evaluating alternative Smac-mimetics, that show encouraging evidence of significant anti-tumor activity, as seen with GDC-0917 as a monotherapy for patients with OC or MALT-lymphoma, HGS1029 against colon cancer and Debio1143 against melanoma metastases [418]. From the perspective of a combined therapeutic, the effects of Smac-mimetics can be enhanced further upon the co-stimulation of cells with death inducing ligands such as TNF-α and TRAIL, and is also an area being developed through inducing their expression with oncolytic viruses and immunomodulatory adjuvants [422].

### Venetoclax (ABT-199)

As the most promising therapeutic from the BH3-mimetics group in targeting the Bcl-2-BAX or -BAK axis of apoptosis regulation, to date 331 clinical trials have been registered testing Venetoclax, and of which 27 have been completed (www.clinicaltrials.gov). As seen in Tables 12, 14 studies have been published in detail, where Venetoclax was seen to exhibit a relatively good safety profile, leading to its evaluation in trials with combined agents and in patients with specific genetic biomarker aberrations.

**Table 14:** Selected phase I-III clinical trials conducted with Venetoclax (V) as a single (−single-) or combined therapeutic in untreated/pre-treated or non-refractory(−)/refractory patients (Refrac) with Mivebresib (Miv), Rituximab and Cyclophosphamide, Doxorubicin, Vincristine, and Prednisone (R-CHOP), Bendamustine (BEND) Rituximab (RIT) Obinutuzumab (OBIN), Cytarabine (CYT), Ibrutinib (IBU) Rituximab (RIT), Bortezomib (BORT) and Dexamethasone (DEX) against Acute Myelogenous Leukemia (AML), Large B-cell Lymphoma (L-BCL), Follicular non-Hodgkin’s Lymphoma (FnHL), Chronic Lymphocytic Leukemia (CLL), Non-Hodgkin’s Lymphoma (NHL), Multiple Myeloma (MM). Objective Response Rates (ORR), Complete Responses (CR) and Partial Responses (PR), expressed as percentage responders (%) are highlighted for disease progression (R/R) patients against R/R with 1/L (1 year treatment) patients. The clinical trials reference numbers highlighted in bold (left column) and the corresponding references highlighted in the column on the right (Ref). Studies where patients were profiled (and their numbers) are highlighted in the Biomarkers column as percentages (%) and ‘-‘indicates no profiling

Venetoclax	Phase	Combined	Patients	Refrac	Disease	ORR, CR, PR	Biomarkers	Ref
**NCT02391480**	I/II	Miv	Pre-treated	Yes	AML	6.66% CR; 6.66% PR	HEXIM1, DCXR, ITD/TKD, PTPN11	[423, 424]
**NCT02055820**	II	R-CHOP	Pre-treated	–	L-BCL	–	Bcl-2, MYC	[425] [426]
**NCT02187861**	II	BEND(B)/ RIT (R)	Untreated (28 d)	Yes	FnHL	75% V + BR; 69% BR (untreated) 4% V + R (non-Refrac) + 19% V + R (Refrac)	Bcl-2/ Mcl1	[427]
**NCT02265731**	II	RIT (R)	Pre-treated	Yes	CLL	100% (V, ORR); 66.7% (V + R, ORR) 16.7% (V, CR) + 50% (V + R, CR)	–	[428]
**NCT01685892**	Ib	OBIN	Untreated	Yes	CLL	95% (R/R, ORR); 100% (1/L, ORR) 37%, (R/R, CR) + 78% (1/L, CR)	IGHV, P53, B2 MG, CD38	[429]
**NCT02287233**	Ib/II	CYT	Untreated	–	AML	62% CR	–	[430]
**NCT02756897**	II	IBRUTINIB	Untreated	–	CLL	88% CR	–	[431]
**NCT01594229**	Ib	BEND/RIT	–	Yes	NHL	65% ORR (30%, CR; 35%, PR)	Bcl-2	[432]
**NCT03755947**	III	RIT	Pre-treated	Yes	17p−/TP53−/IGHV-CLL	92.3% ORR; (17.48%, CR)	–	[433–439]
**NCT01794507**	Ib	BORT/DEX	Pre-treated	Yes	MM	67% ORR	Bcl-2 Bcl-xL, Mcl1,	[440]
**NCT01889186**	II	**-single-**	–	Yes	del17p-CLL	70% ORR	17P del (50.3%)	[441–443]
**NCT01994837**	II	**-single-**	Pre-treated	–	AML	19% ORR (6% CR)	Bcl-2, Bcl-xL, BH3 Profiling, Mcl1	[444]
**NCT01328626**	I	**-single-**	–	Yes	CLL	79% ORR (20% CR)	17Pdel	[435, 443]

As seen, Venetoclax is showing itself to be a very promising therapeutic, against Bcl-2-dependent cancer cells such as CLL, acute leukemias, breast cancer, *myc*-driven lymphomas [293–295], and through successful phase I-III trials for acute myeloid leukemia (AML), CLL, MM and non-Hodgkin’s lymphoma (NHL) [296], it was ultimately awarded US Food and Drug Administration (US FDA) and European Medicines Agency (EMA) approval for treating CLL patients harboring the 17p or p53 mutations [445].

As a mono-therapeutic, Venetoclax has also been reported to be effective against other lymphomas with high Bcl-2 expression levels, as in MCL, myeloma, refractory AML [444], follicular lymphoma and some diffuse large B-cell lymphomas [446], whereby the dose administered could be correlated with expression levels of Mcl1 and Bcl-xL [447]. Over the last 3 years a number of follow-up trials have been published, aimed at defining its use against drug resistance, with some very encouraging outcomes based on its combined use with other therapeutics such as DNA damaging agents, *anti*-CD20 antibodies (such as Rituximab), hyper-methylating agents, kinase inhibitors, Mdm2 inhibitors, proteasome inhibitors, and in conjunction with inhibitors targeting the anti-apoptotic proteins Bcl-xL and Mcl1 [448]. At a time when there are a plethora of on-going clinical trials involving Venetoclax (www.clinicaltrials.gov), its combined use with Rituximab is showing some very promising outcomes with complete remissions recorded for 51% of relapsed CLL patients [449], and it is also leading the way for use with patients encoding specific genetic biomarker aberrations (NCT02391480, NCT02055820, NCT02187861, NCT01685892, NCT01594229, NCT01794507, NCT01994837, NCT01328626), and where serious side effects (such as neutropenia and thrombocytopenia) are being managed with the growth factor prophylaxes (NCT02265731, NCT02055820, NCT01889186). Collectively, such findings encouragingly highlight the potential of Venetoclax as a developing model for the treatment of hematological cancers, and thus paving the way for its further testing on solid tumors.

### Mcl1-inhibitors

Although genetically elevated Mcl1 levels have been reported in many tumor types [26], it has been implicated in therapeutic resistance of breast and lung cancers [450, 451], with some studies showing that Mcl1 expression can mediate resistance to Navitoclax or Venetoclax [70, 99, 452], Gemcitabine, Vincristine and Taxols [453–455]. To date 36 clinical trials have been registered utilizing Mcl1 inhibitors (www.clinicaltrials.gov.uk), amongst which 10 have been completed, 2 withdrawn, 4 terminated and 8 are still recruiting. Of the completed trials related to targeting the Mcl1 protein (Table 15), S64315 has been tested as a single agent against MM and DLBCL (NCT02992483) in untreated relapsed/refractory patients to establish maximum tolerated doses and the results for which are to be published.

**Table 15:** Selected phase I clinical trials conducted with MIK655 or S64315 as a single (S) therapeutic, on untreated, and refractory (Refrac) diseases, such as Multiple Myeloma (MM), Diffuse Large Cell B-Lymphoma (DLBCL), Acute Myeloid Leukemia (AML) and Myelodysplastic Syndrome (AST) patients, for Maximum Tolerated Dose (MTD) are highlighted. Adverse effects (Adv. Effects) are highlighted as Nausea (Nau), Familial Neutropenia (F-Neut) and Diarrhoea (Diar). The clinical trials reference numbers are highlighted in bold (left column) and the Objective Response Rates (ORR) expressed as the percentage of patients who responded positively (%). Unavailable data is highlighted by ‘-‘

MIK655/S64315	Phase	Single	Patients	Refrac	Disease	Outcomes	Adv. Effects	ORR
**NCT02992483**	I	S	Untreated	Yes	MM, DLBCL	MTD	–	Unpublished
**NCT02979366**	I	S	Untreated (14 days)	Yes	AML, MDS	MTD	Nau/F-Neut/Diar	0% ORR

Similarly, trials for S64315 efficacy have also been conducted in AML and MDS patients, and which showed no significant benefits outside of side effects such as nausea and familial neutropenia (NCT02979366). Consequently, concerns regarding the safety of Mcl1 inhibition as a single agent, have arisen and are founded on the dependency of Mcl1 expression for the normal survival and growth of cells, as seen for mouse cardiomyocytes [456], hepatocytes [457] and neurons [458]. In support of this viewpoint, Mcl1^+/−^ mice should mimic a phenotype consistent with 50% inhibition of Mcl1, but they appear normal and healthy, thus supporting the belief that Mcl1 may also function independently of BH3-domain protein binding and regulation during cellular death [321, 459]. Another challenge for the development of specific Mcl1 inhibitors has emerged from the rigidity of the Mcl1 protein hydrophobic pocket and competitiveness for this posed by alternative high-affinity endogenous protein binding partners [460]. Nevertheless, several highly potent (sub-nanomolar) and selective inhibitors have emerged very recently, and one of which (UMI-77) shows excellent potential against pancreatic cell lines in vitro and in xenograft pre-clinical models [461]. Similarly, pre-clinical findings for the inhibitor S63845 have been very impressive [74], against several cancer types including melanoma, leukemia, lymphoma, and other solid cancers either as a single or combined agent, and further work here will indeed help in extending these findings towards clinical studies.

In summary, while serious side effects and solubility issues had been reported for the founding therapeutic member ABT-737, as seen from the findings presented in Tables 10, 11, 12, 13, 14, 15, derivatives of this appear to be showing incredible promise in treating hematological and solid cancers. With concerted efforts being made in the clinic to address side effects, the members that are showing steady progress from phases I to III, include Venetoclax and Obatoclax, with progress from studies evaluating Mcl1- and Smac- mimetics (or inhibitors) maintaining steady momentum. When coupled with evaluating patients for genetic biomarker mutation profiling, a very clear and interesting picture is coming into focus with regards to how some of these mimetics may have greater efficacy and potential in a personalized medicine context, and as encouragingly seen from clinical trials highlighted in Table 14.

### Mimetics: current considerations for solubility and delivery

In light of the above developing areas for therapeutic mimetics, key areas for therapeutic delivery have also been given greater consideration, particularly in response to solubility and delivery limitations arising from the ABT-737 paradigm. Classically, while therapeutic affinity for the target protein is of paramount importance when designing an intervention strategy, additional factors affecting therapeutic availability, longevity, toxicity, and penetrability are also being given greater collective importance. In the next section, we selectively outline and evaluate the recent progress that has been reported in the areas of SMI and peptide therapeutics delivery, with a view to highlighting their usefulness for mimetic bioavailability and solubility, which (from the ABT-737 paradigm) were serious considerations that hindered the immediate evaluation of such a promising therapeutic.

As many SMIs are hydrophobic, which may present solubility and bioavailability challenges, the recently demonstrated successes of cell-penetrating peptide (CPP) nanoparticles [462] or nanofibers [463, 464] are emerging as synergistic alternatives for therapeutic solubilization and the delivery of therapeutic cargo. Briefly, the combined use of monomeric CPP amphiphilic peptides with cholesterol can self-assemble, forming a shell-structured nanoparticle, that contains a hydrophobic cholesterol core and a hydrophilic cationic exterior, which can enhance solubility, loading, delivery, and uptake of potential therapeutics [462]. Similarly, the potential of supramolecular nanofiber technology in this context is evidenced through them being able to enhance the incorporation of Paclitaxel from 6.8 to 41% [465], from a self-assembling amphiphilic peptide monomer [466]. Moreover, such applications can be extended to incorporate *anti*-inflammatories [467], multi-drug combinations [468] and imaging agents [469, 470], which collectively highlight the potential of such approaches.

While, CPPs can enhance cargo delivery of drugs using nanostructures [462, 471, 472], reduced specificity, resulting toxicity and reduced half-life can present challenges, as with most delivery systems of this nature [473, 474]. In the context of delivering BH3-mimetics, one good example which demonstrates how some of these issues of specificity and delivery can be overcome, was published by Schnorenberg et al. (2019), who utilized a BIM BH3-mimetic peptide amphiphile nanostructure to facilitate uptake for triggering the death of Hela and MEF cells [475]. Interestingly, a cathepsin B cleavage signal was also incorporated between the mimetic and hydrophobic tail to permit release of the mimetic at the site of enhanced ECM turnover, or within cells following endosomal and lysosomal uptake [475].

Importantly, the use of supra-molecular peptide delivery systems is being seen to offer certain advantages over classical delivery systems, such as high-concentration delivery of peptides, stabilization of peptide secondary structure, protection from proteolysis within the circulatory system, increased half-life, delivery of multiple therapeutics and enhanced uptake to the endosomal and lysosomal compartments [476, 477]. While carrier peptides or CPPs can be utilized to help aide solubility and penetrability, an alternative targeting approach worth highlighting is the use of Elastin-like polypeptides (ELPs), which can form aggregates when heated externally and which can selectively accumulate in tumors [478–480]. Such an approach exploits the increased permeability of tumor vasculature during regional hyperthermia, during which a greater accumulation of macromolecular drug carriers loaded with single or combined therapeutics is permitted [481–483]. This permits enhanced cytotoxicity [484], which can be optimised further through CPP tagging for delivery to cells in vitro or in vivo [485–488]. While some peptides may require certain conformations to achieve full inhibitory activity or cellular penetrability, pH dependent membrane insertion (pHLIPS) may offer additional potential benefits, from the perspective of correcting structurally disorganized peptide therapeutics to adopt a favorable α-helical structure during their delivery and thereafter [489–491].

Lastly, the use of phospholipids [492] for self-assembling micelles and the delivery of safe and non-immunogenic, hydrophobic drugs such as cyclosporine A [493], Paclitaxel [494] and peptides [495], can minimize the degradation or aggregation of cargo [496–499]. Encouragingly, such approaches can enhance therapeutic efficacy through reduced extravasation from the circulation, while simultaneously reducing adverse off-target effects [495, 500]. However, such an approach does come with limitations that cannot go ignored, due to potentially diminished availability of the helical peptide therapeutic moiety, through masking and immunogenic effects from PEG, or limitations in peptide cargo lengths of 17–36 aa [501, 502]. Nevertheless, as a way of highlighting the potential of this approach, Gossypol loaded micelles combined with A549 cells and radiation therapy in a xenograft mouse model [503], showed 7x greater efficacy against tumors than Gossypol alone [504]. Moreover, biomimetic poly(lactic-coglycolic acid) (PLGA) combined with red blood-cell membrane (RBCm) as nanoparticles loaded with Obatoclax mesylate for the treatment of non-small-cell lung cancer, also showed significant success in prolonging drug circulation in the presence of a reduced immune response against the particles [505].

## Conclusions

In summary, while ABT-737 was discovered as an authentic BH3-mimetic, derivatives such as Navitoclax and Venetoclax hold great potential in treating hematological cancers and even solid tumors when administered with classical and conventional anti-cancer drugs, and the evidence for which is firmly grounded on basic research efforts at the pre-clinical level. In this context, Smac-mimetics have also emerged as being promising candidates for treating a range of cancer types, as seen from their combined compatibility with a broader range of anti-cancer drugs in pre-clinical models. However, the most promising potential therapeutics in pre-clinical models appear to be the Mcl1 inhibitors, particularly when administered in combination with Venetoclax, Navitoclax, death inducing ligands and conventional anti-cancer drugs.

Through extending such findings by way of clinical trials, the therapeutic efficacy of the most promising anti-cancer drugs are coming into fruition, either as single or as combined agents. While resistance has always served to hinder the development of many therapeutics for cancer, the off-target effects of such agents are becoming increasingly clear, and in some instances are also being harnessed through the development of dual-activity inhibitors, or as agents that can be synergistically utilized to overcome any resistance effects. From the above findings, the best example of this is highlighted by the effectiveness of Mcl1 inhibitors, when combined with the drugs Navitoclax or Venetoclax as co-therapeutics, and the findings from which are also being effectively aligned with specific genetic biomarkers of interest. Lastly, of emerging interest, in this context, are the BH3-mimetics Obatoclax and Gossypol, and Smac-mimetics, the clinical importance of which are eager awaited.

As the focus of this article has revolved around the regulation of the intrinsic arm of apoptosis, the effects of the above mimetics and inhibitors towards other cell death-inducing off-target effects, cannot be ignored. Such an assertion arises from what contribution these therapeutics may offer in minimizing the protective effects of pro-survival proteins, associated with the ER stress-response [403, 506], autophagy [507, 508], necroptosis [406] or cell cycle deregulation [509, 510]. When considered alongside how technologies have developed to improve solubility and the delivery of therapeutics, and thereby improving their efficacy, the initial limitations surrounding candidates (such as ABT-737), based on lack of solubility and availability, can be addressed with greater optimism. Such developments indeed offer greater certainty in pursuing mimetics as therapeutics and which follow a strongly-validated paradigm. From an equally important perspective, the presence of clinical side effects, such as thrombocytopenia have always appeared as a major source of concern. As mentioned, such effects can be minimized through molecular approaches, as in the instance of re-engineering Navitoclax to DT2216, and which acts through degrading Bcl-xL [383], thus reducing its off-target effects. Moreover, complimentary approaches through the use of prophylaxes are also being implemented, as effective management strategies within the clinic.

When considered together, the previous 20-30 years of mimetics research have indeed come a long way, whilst occupying a vast area of development in cancer treatment, and which lay very strong technological foundations for the future development of novel single-, combined- or dual- action therapeutics, with personalized medicine being well positioned at the forefront of such endeavors.

## Data Availability

All data and materials cited herein are available upon request.

## Abbreviations

ALL: Acute lymphoblastic leukaemia
AML: Acute myelogenous leukemia
BAX: Bcl-2-associated protein X
BAK: Bcl-2 antagonist/killer
BC: Breast Cancer
BOK: Bcl-2 ovarian killer
Bcl-2: B cell lymphoma-2
Bcl-xL: B cell lymphoma extra-large
BH domain: Bcl-2 homology domain
Bid: Bcl-2 interacting domain death agonist
Bim: Bcl-2 interacting mediator of cell death
BIR: Baculovirus IAP Repeat
CAD: Caspase-activated DNAase
CLL: Chronic lymphocytic leukaemia
CPP: Cell penetrating peptide
DLBCL: Diffuse large B-cell lymphoma
ER: Endoplasmic reticulum
HNSCC: Head and neck squamous cell carcinoma
Mcl1: Myeloid cell leukemia-1 protein
EGF-R: Epidermal growth factor-receptor
GC: Gastric Cancer
cIAP: Cellular inhibitor of apoptosis
MCL: Mantle cell lymphoma
MPC: Mitochondrial pore complex
MOMP: Mitochondrial outer membrane permeabilization
NF-κB: Nuclear factor kappa B
NHL: Non-Hodgkin’s lymphoma
NSCLC: Non-small cell lung cancer
MM: Multiple myeloma
PARP: Poly ADP-ribose polymerase
PC: Prostate Cancer
PROTAC: Proteolysis targeting chimera
pHLIPS: pH-Low Insertion Peptide
Puma: p53-upregulated modulator of apoptosis
SAHBs: Stabilized alpha-helix of BCL-2
SCLC: Small cell carcinoma
Smac/DIABLO: Second mitochondria-derived activator of caspases
SMI: Small molecule inhibitor
T-ALL: T-cell acute lymphoblastic leukaemia
TNF-α: Tumor necrosis factor-alpha
TRAIL: Tumor necrosis factor-related apoptosis-inducing ligand
